# Pyronaridine as a Bromodomain-Containing Protein 4-*N*-Terminal Bromodomain (BRD4-BD1) Inhibitor: *In Silico* Database Mining, Molecular Docking, and Molecular Dynamics Simulation

**DOI:** 10.3390/molecules28155713

**Published:** 2023-07-28

**Authors:** Mahmoud A. A. Ibrahim, Mahmoud M. H. Abdelhamid, Khlood A. A. Abdeljawaad, Alaa H. M. Abdelrahman, Gamal A. H. Mekhemer, Peter A. Sidhom, Shaban R. M. Sayed, Paul W. Paré, Mohamed-Elamir F. Hegazy, Tamer Shoeib

**Affiliations:** 1 Computational Chemistry Laboratory, Chemistry Department, Faculty of Science, Minia University, Minia 61519, Egypt; 2 School of Health Sciences, University of KwaZulu-Natal, Westville Campus, Durban 4000, South Africa; 3 Department of Pharmaceutical Chemistry, Faculty of Pharmacy, Tanta University, Tanta 31527, Egypt; 4 Department of Botany and Microbiology, College of Science, King Saud University, P.O. Box 2455, Riyadh 11451, Saudi Arabia; 5 Department of Chemistry & Biochemistry, Texas Tech University, Lubbock, TX 79409, USA; 6 Chemistry of Medicinal Plants Department, National Research Centre, 33 El-Bohouth St., Dokki, Giza 12622, Egypt; 7 Department of Chemistry, The American University in Cairo, New Cairo 11835, Egypt

**Keywords:** cancer disease, bromodomain-containing protein 4, SuperDRUG2 database, molecular docking, molecular dynamics simulations

## Abstract

BRD4 (bromodomain-containing protein 4) is an epigenetic reader that realizes histone proteins and promotes the transcription of genes linked to cancer progression and non-cancer diseases such as acute heart failure and severe inflammation. The highly conserved *N*-terminal bromodomain (BD1) recognizes acylated lysine residues to organize the expression of genes. As such, BD1 is essential for disrupting BRD4 interactions and is a promising target for cancer treatment. To identify new BD1 inhibitors, a SuperDRUG2 database that contains more than 4600 pharmaceutical compounds was screened using *in silico* techniques. The efficiency of the AutoDock Vina1.1.2 software to anticipate inhibitor-BRD4-BD1 binding poses was first evaluated based on the co-crystallized R6S ligand in complex with BRD4-BD1. From database screening, the most promising BRD4-BD1 inhibitors were subsequently submitted to molecular dynamics (MD) simulations integrated with an MM-GBSA approach. MM-GBSA computations indicated promising BD1 binding with a benzonaphthyridine derivative, pyronaridine (SD003509), with an energy prediction (Δ*G*_binding_) of −42.7 kcal/mol in comparison with −41.5 kcal/mol for a positive control inhibitor (R6S). Pharmacokinetic properties predicted oral bioavailability for both ligands, while post-dynamic analyses of the BRD4-BD1 binding pocket demonstrated greater stability for pyronaridine. These results confirm that *in silico* studies can provide insight into novel protein–ligand regulators, specifically that pyronaridine is a potential cancer drug candidate.

## 1. Introduction

It is known that 46 proteins in the human body contain 61 isoforms of bromodomains (BRDs), which can be categorized into eight groups and have about 140 amino acid residues in their sequences [1,2]. According to earlier studies, BRDs have a highly conserved fold structure that predominantly consists of two flexible loops (called the ZA-loop and BC-loop), which are responsible for substrate specificity, and four *α*-helices, described as *α*A, *α*B, *α*C, and *α*Z [3,4]. The bromodomain and extra terminal domain (BET) family consists of four members known as BRD2, BRD3, BRD4, and BRDT. BRD4 (bromodomain-containing protein 4), an epigenetic reader, is one of the most important BET proteins. BRD4 has a fundamental function in angiogenesis and subsequent health-related disorders, ranging from inflammation-associated diseases to carcinoma diseases. Similar to the remaining BET family members, BRD4 involves BD1 and BD2, as well as extra-terminal (ET) domains [5]. BD1 and BD2 identify lysine residues to arrange cycle progression and apoptosis [6,7,8,9]. Both BDs are extremely preserved and symmetrical [2]; each participates in protein-specific binding differently and possesses different biological roles because of their interactions with various types of proteins [1]. BRD4-BD1 demonstrates a greater affinity for the H4 (tetra-acetylated histone) peptide. However, BRD4-BD2 relates to H3 (diacetylated histone) [10].

Several inhibitors for BET are being utilized in clinical investigations, including azepine BET inhibitors with nanomolar IC_50_ values [3,11,12]. (+)-JQ1 is the BET family’s first and most well-studied inhibitor and is capable of inhibiting both the BD1 and BD2 of BRD4 [13,14,15,16,17,18]. BET inhibitors have limited selectivity among proteins within the family. Olinone is a BRD4-BD1-selective inhibitor [19], but MS765 and RVX-208 inhibit BD2 more selectively than BD1 [20,21]. Selective inhibitors allow for the differentiation of specific BD2 and BD1 roles in specific illnesses. According to a recent study, BRD4-BD1 can maintain oncogene expression in established transcriptional pathways, in contrast to the essentially ineffectual BRD4-BD2. However, no drug has yet been approved as a BRD4-BD1 inhibitor. Therefore, there is still a demand to explore the potentialities of other active pharmaceutical ingredients against BRD4-BD1.

**Figure 1:**
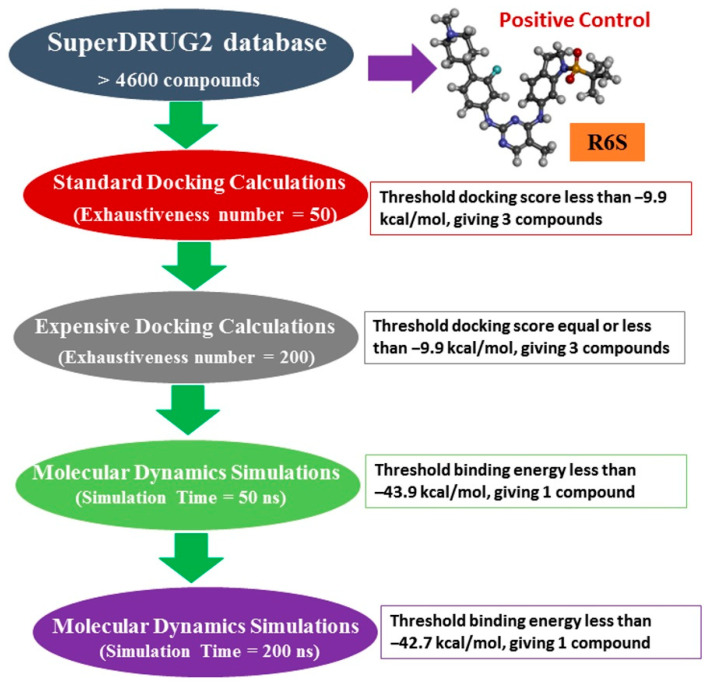
Flowchart diagram of the employed computational methods and filtration process for the SuperDrug2 database.

Herein, the SuperDRUG2 database, which contains >4600 pharmaceutical compounds, was screened against BRD4-BD1. Molecular docking was utilized to filter the database, and the most potent BRD4-BD1 inhibitors were undergone MD simulations. The inhibitor-BRD4-BD1 binding energy was evaluated utilizing the MM-GBSA approach. Ultimately, physicochemical and ADMET features were predicted for the most promising drug candidates. The workflow of the utilized *in silico* methods is shown in Figure 1. This study identified a compound with a promising inhibitor against BRD4-BD1, and additional experimental investigations are recommended.

## 2. Results and Discussion

BRD4 has attracted special focus in treating carcinoma because of its vital role in regulating oncogene expression [22]. Bromodomain-1 (BRD4-BD1) activity is primarily related to carcinoma and can preserve oncogene expression in established transcriptional pathways [23]. The inhibition of BRD4-BD1 is considered a good target for treating cancer. Because no drug has yet been approved as a BRD4-BD1 inhibitor, exploring potential drug candidates against BRD4-BD1 is needed [24]. Consequently, the SuperDRUG2 database was examined and filtered to identify putative BRD4-BD1 inhibitors.

### 2.1. Docking Protocol Validation

**Figure 2:**
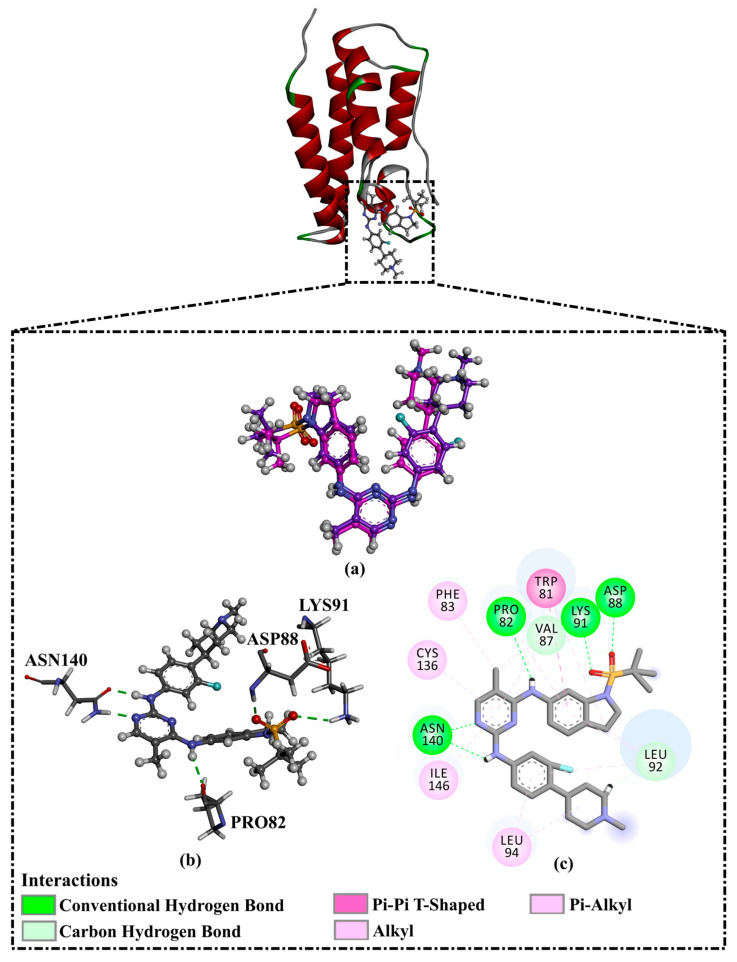
(**a**) Three-dimensional superimpositions of the docked pose (in pink) and co-crystallized pose (in mauve) of R6S; (**b**) three-dimensional depictions of intermolecular hydrogen bond interactions; and (**c**) two-dimensional depictions of noncovalent interactions for the anticipated docking mode of R6S inside the BRD4-BD1 binding pocket.

Prior to data generation, the employed docking protocol and parameters for AutoDock Vina1.1.2 were validated in accordance with the experimental data. The co-crystallized R6S inhibitor was re-docked toward the BRD4-BD1 binding pocket, and the anticipated binding mode was compared with its native structure (PDB ID: 7REK [25]). Figure 2 illustrates that the predicted docking mode was almost the same as the native structure of R6S inside the BRD4-BD1 binding pocket, with an RMSD value of 0.82 Å. The binding pose displayed five crucial hydrogen bonds (Figure 2). In detail, the NH groups of the R6S inhibitor displayed two H-bonds with ASN140 (2.71 Å) and PRO82 (3.04 Å) (Figure 2). The nitrogen atom of the pyrimidine ring established an H-bond with the NH_2_ group of ASN140 (1.92 Å). Additionally, the (methylsulfonyl)methane group demonstrated two H-bonds with the NH group of ASP88 (1.93 Å) and the NH_3_ group of LYS91 (2.45 Å) (Figure 2). Notably, the aromatic benzene ring of R6S exhibited a π-π T-shaped interaction with TRP81 (Figure 2). Based on the re-docking results, the AutoDock Vina1.1.2 software correctly predicted the proper binding mode of inhibitor-BRD4-BD1. Therefore, the AutoDock Vina1.1.2 software was utilized to virtually screen the SuperDRUG2 database to hunt for prospective BRD4-BD1 inhibitors.

### 2.2. Virtual Screening of the SuperDRUG2 Database

**Figure 3:**
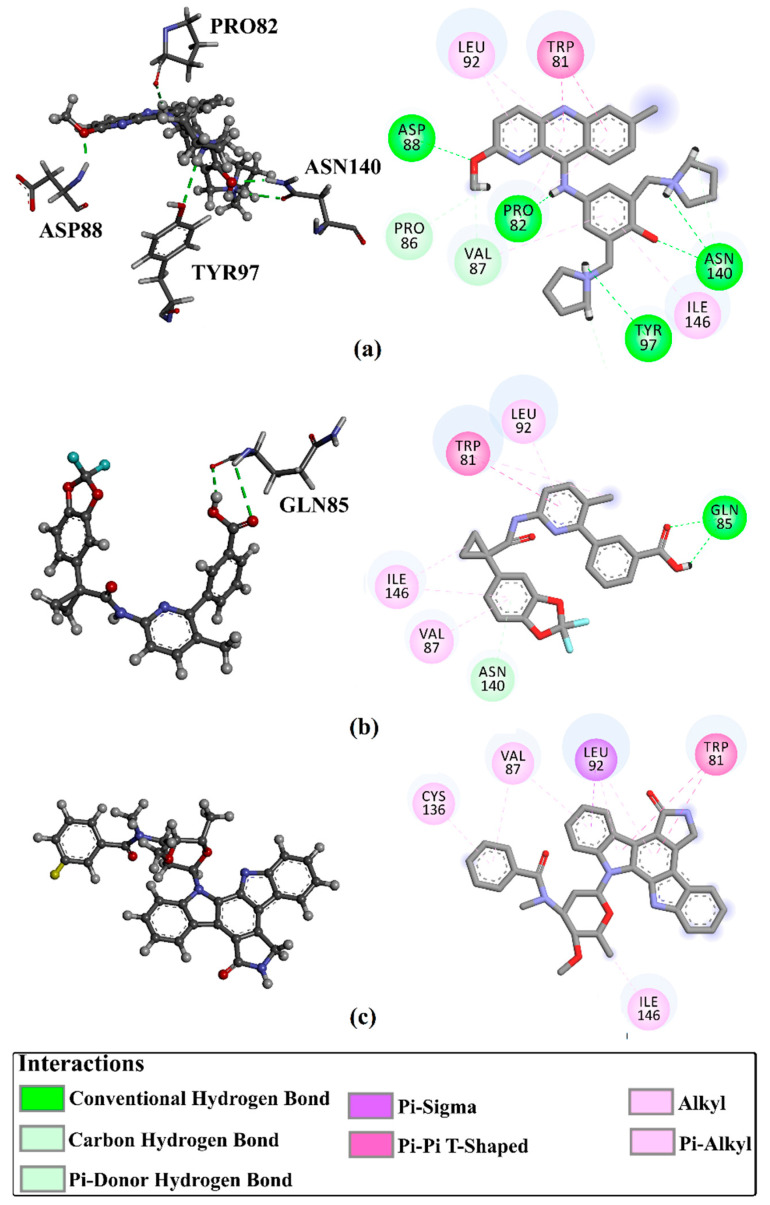
Three-dimensional depictions of intermolecular hydrogen bond interactions and two-dimensional depictions of noncovalent interactions for (**a**) pyronaridine (SD003509), (**b**) lumacaftor (SD003873), and (**c**) *N*-benzoylstaurosporine (SD006001) within the BRD4-BD1 binding pocket.

Virtual screening is a valid technique for rapidly identifying potential bioactive inhibitors during the premature stages of drug discovery [26,27]. The SuperDRUG2 database was virtually screened against BRD4-BD1 by utilizing two stages of molecular docking computations. Initially, standard docking calculations were performed for all inhibitors in the database against BRD4-BD1 with an exhaustiveness number of 50, and the docking scores were predicted (Table S1). Based on the standard docking scores, only three inhibitors had docking scores similar to or less than that of R6S (calc. −9.9 kcal/mol). Consequently, these inhibitors were picked out for expensive docking calculations with an exhaustiveness number of 200. Table 1 summarizes the binding features, docking scores, and two-dimensional chemical structures of the three inhibitors. In addition, the binding poses are illustrated in Figure 3. As demonstrated, the three inhibitors exhibited the same binding poses within the BRD4-BD1 binding pocket.

Pyronaridine (SD003509), a benzonaphthyridine derivative, has been identified as a potential anti-malaria drug candidate [28]. Pyronaridine had the lowest docking score with BRD4-BD1 (calc. −10.2 kcal/mol), forming five hydrogen bonds against BRD4-BD1 (Table 1 and Figure 3). The docking pose of pyronaridine-BRD4-BD1 showed that the NH of the 1-methylpyrrolidin-1-ium group formed two H-bonds with ASN140 (2.32 Å) and TYR97 (2.67 Å). In addition, the NH of the dimethylamine demonstrated an H-bond with PRO82 (2.23 Å). Finally, the 6-methoxy-2,3-dihydropyridine group formed an H-bond with the NH of ASP88 (1.95 Å) (Table 1 and Figure 3). Notably, the pyronaridine inhibitor demonstrated a π-π T-shaped interaction with TRP81 and π-alkyl interactions with PRO82, VAL87, LEU92, and ILE146 (Table 1 and Figure 3).

Lumacaftor (SD003873) is an aromatic amide used to treat cystic fibrosis (CF) [29,30]. Lumacaftor also displayed a good docking score toward the BRD4-BD1 protein (calc. −10.1 kcal/mol) (Table 1). Investigating the docking pose of lumacaftor inside the BRD4-BD1 binding pocket showed that the carboxylic group demonstrated two H-bonds with the acetamide group of GLN85 (2.45 and 3.08 Å) (Table 1 and Figure 3). Additionally, lumacaftor exhibited π-π T-shaped interactions with TRP81 and π-alkyl interactions with VAL87, TRP81, LEU92, and ILE146 (Table 1 and Figure 3).

*N*-benzoylstaurosporine (SD006001), an oral inhibitor of protein kinase C, has the potentiality for cell cycle targeting [31]. *N*-benzoylstaurosporine also revealed a favorable docking score against BRD4-BD1 (calc. −10.0 kcal/mol). The docking pose of *N*-benzoylstaurosporine did not show any H-bonds with the critical residues of the BRD4-BD1 binding pocket. For the π-based interactions, the 3,7-dihydro-2H-indole group exhibited two π-alkyl interactions with the alkyl group of LEU92 (4.50 and 4.99 Å) (Table 1 and Figure 3). In addition, the two benzene rings formed two π-π T-shaped interactions with the alkyl group of VAL87 with bond lengths of 4.86 and 5.22 Å. Furthermore, the benzene ring of the 3,7-dihydro-2H-indole group interacted through π-alkyl with CYS136 (Table 1 and Figure 3).

Notably, the interactions of docked structures of pyronaridine, lumacaftor, and *N*-benzoylstaurosporine confirmed the great significance of π-alkyl, hydrogen bonding, and π-π T-shaped interactions with the fundamental residues of the BRD4-BD1 binding pocket in the eminent docking scores of these inhibitors.

### 2.3. Molecular Dynamics (MD)

To assess the docking results and gain more insight into the constancy of the inhibitor-receptor complexes, MD simulations were executed [32,33]. MD simulations were conducted for the three promising inhibitors complexed with the BRD4-BD1 protein for 50 ns. Furthermore, the binding energies were computed utilizing the MM-GBSA approach (Table 2). The data imply that only pyronaridine exhibited a binding energy (Δ*G*_binding_) of −46.2 kcal/mol, which is smaller compared with that of the R6S inhibitor (calc. −43.9 kcal/mol). However, lumacaftor and *N*-benzoylstaurosporine showed low binding affinities with Δ*G*_binding_ of −27.8 and −20.0 kcal/mol, respectively (Table 2). To obtain more reliable results, MD simulation for pyronaridine complexed with the BRD4-BD1 protein was prolonged to 200 ns, followed by a binding energy estimation (Table 2). Pyronaridine showed promising binding affinity throughout the 200 ns MD simulations, with a Δ*G*_binding_ of −42.7 kcal/mol in comparison with −41.5 kcal/mol for R6S complexed with the BRD4-BD1 binding pocket (Table 2). For more reliable results, the MD simulation was repeated two more times for pyronaridine-BRD4-BD1 complex over 200 ns MD simulations, followed by binding energy computations. Pyronaridine unveiled almost identical binding affinities in the duplicates, with a Δ*G*_binding_ of −42.1 and −43.7 kcal/mol. An MM-GBSA binding energy comparison of pyronaridine with R6S demonstrated competing binding affinities, suggesting the *in silico* potentiality of the two compounds as BRD4-BD1 inhibitors. These findings demonstrated that pyronaridine and RS6 are promising BRD4-BD1 inhibitors and may act as prospective drug candidates for cancer remediation.

**Figure 4:**
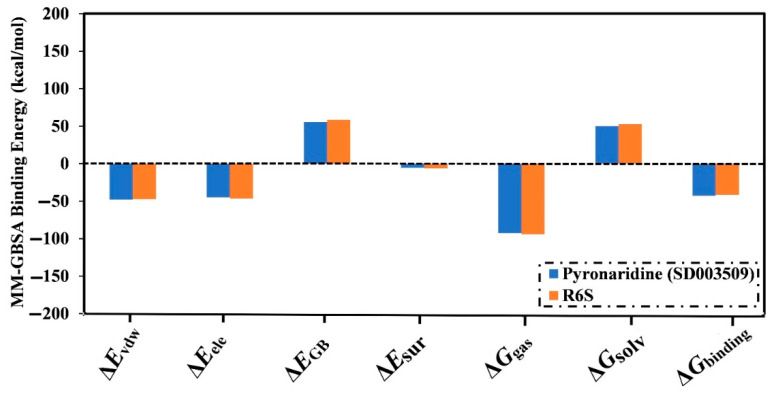
Binding energy decomposition for pyronaridine (SD003509) and R6S inhibitor complexed with the BRD4-BD1 protein over a 200 ns MD simulation.

The decomposition of binding energies was carried out to reveal the type of interactions that are dominating the binding of pyronaridine within the BRD4-BD1 binding pocket, as presented in Figure 4. The *E*_vdW_ energy significantly contributed to the pyronaridine- and R6S-BRD4-BD1 binding affinities (calc. −47.9 and −47.5 kcal/mol, respectively). Furthermore, *E*_ele_ also favorably contributed with values of −44.8 and −46.6 kcal/mol for the pyronaridine- and R6S-BRD4-BD1 complexes, respectively.

**Figure 5:**
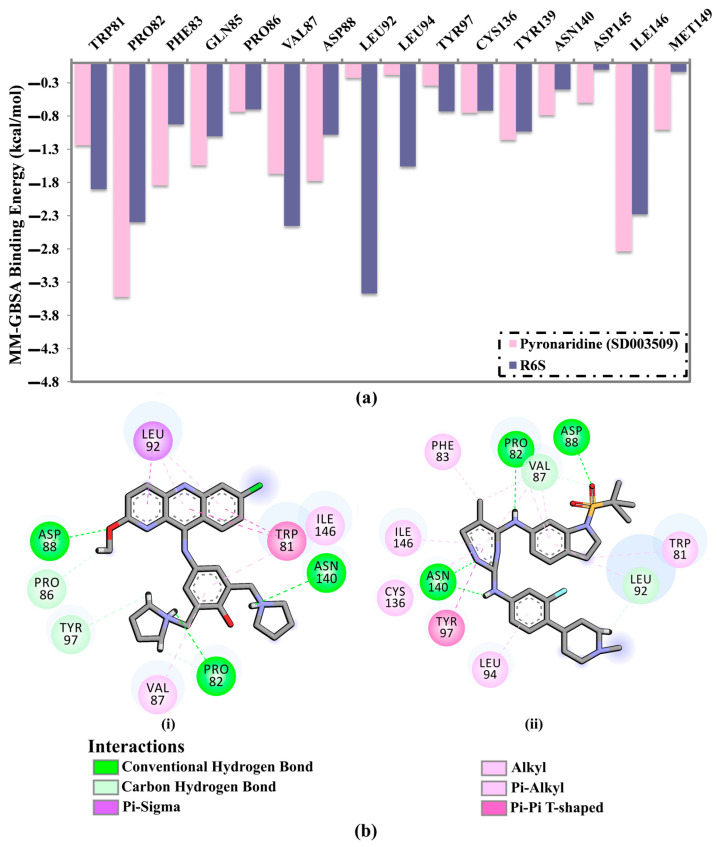
(**a**) Per-residue energy participation of the critical residues (in kcal/mol) and (**b**) two-dimensional depiction of the binding modes of (i) pyronaridine (SD003509) and (ii) the R6S inhibitor with the BRD4-BD1 protein based on the average structure throughout the MD simulation.

To find the proximal amino acids inside the BRD4-BD1 protein that participate in interactions with the inhibitor, per-residue energy decomposition was performed for the pyronaridine and R6S complexed with BRD4-BD1 during the 200 ns MD simulation. Figure 5a shows the energy contributions of the residues with binding energy < −0.5 kcal/mol. The PRO82, VAL87, ASP88, ASN140, and ILE146 residues significantly contributed to the interactions between the BRD4-BD1 protein and pyronaridine and R6S (Figure 5a). For instance, PRO82 contributed substantially to ∆*G*_binding_ with values of −3.5 and −2.4 kcal/mol for pyronaridine and R6S complexed with BRD4-BD1 protein, respectively (Figure 5a). In addition, both TRP81 and ILE146 had a significant impact on the binding of pyronaridine and R6S with BRD4-BD1 protein (Figure 5a).

The average structures of pyronaridine and R6S within the BRD4-BD1 protein over the 200 ns MD simulations are also represented (Figure 5b). The average structure displayed a similar binding mode to the docked structure of pyronaridine, forming three H-bonds with BRD4-BD1 critical residues over the 200 ns MD simulation. Notably, a H-bond with TYR97 in the docked pyronaridine-BRD4-BD1 complex was absent in the average structure (Figure 5b). This demonstrates the significance of conducting MD simulations to obtain trustworthy results.

### 2.4. Post-MD Analyses

In order to evaluate the consistency of pyronaridine- and R6S-BRD4-BD1 complexes, post-MD analyses were carried out throughout the 200 ns MD simulation and compared with those of the co-crystallized R6S inhibitor.

#### 2.4.1. Binding Energy Per Trajectory

**Figure 6:**
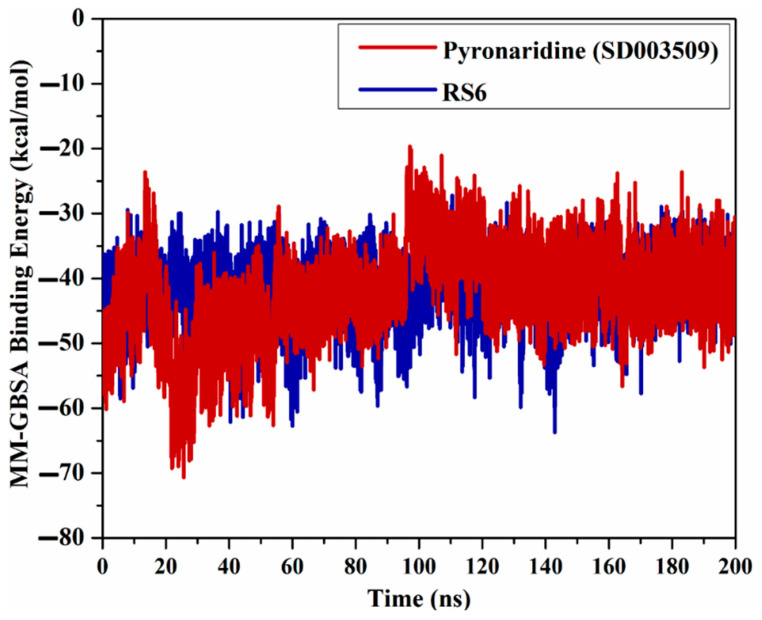
Variations in the binding energies for pyronaridine (SD003509) (in dark red) and R6S inhibitor (in navy) against the BRD4-BD1 protein throughout the MD simulation.

A binding energy vs. time analysis was conducted to investigate the steadiness of pyronaridine and R6S inhibitors within the BRD4-BD1 binding pocket (Figure 6). According to Figure 6, pyronaridine and R6S displayed excellent stability, with ∆*G*_binding_ values of −42.7 and −41.5 kcal/mol, respectively. These outcomes prove the steadiness of pyronaridine- and R6S-BRD4-BD1 complexes over the simulation time.

#### 2.4.2. H-Bond Analysis

**Figure 7:**
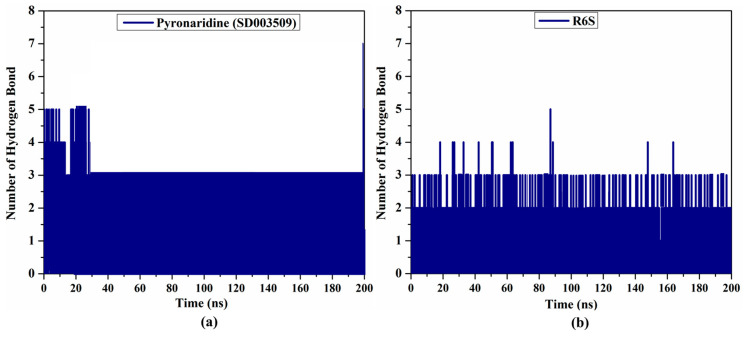
Number of H-bonds established between (**a**) pyronaridine (SD003509) and (**b**) R6S and the BRD4-BD1 protein throughout the MD simulations.

During the 200 ns MD simulations, an H-bond analysis between the inhibitor and BRD4-BD1 protein was utilized to evaluate the interaction steadiness. The number of H-bonds established between the pyronaridine/R6S and BRD4-BD1 protein in the collected snapshots was computed (see Figure 7). There were three estimated H-bonds for both the pyronaridine- and R6S-BRD4-BD1 complexes. Overall, these results indicate a good agreement with the average structures illustrated in Figure 5b.

#### 2.4.3. Center-of-Mass Distance (CoM)

**Figure 8:**
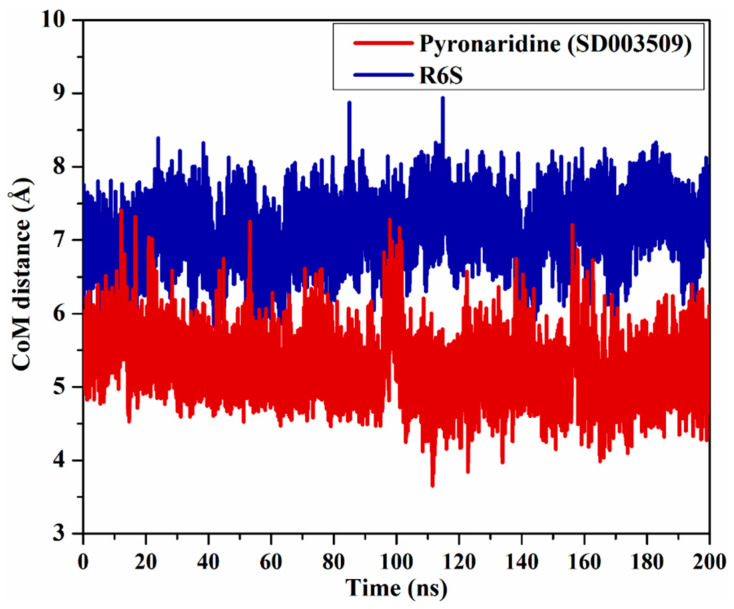
CoM distance between pyronaridine (SD003509) (in dark red) and the R6S inhibitor (in navy) and PRO82 inside the BRD4-BD1 active site throughout the MD simulation.

The CoM distance between pyronaridine/R6S and PRO82 was investigated to better understand the inhibitor’s stability within the binding pocket of the BRD4-BD1 protein (Figure 8). According to the CoM data in Figure 8, the measured CoM distance remained steady for pyronaridine and R6S with the BRD4-BD1 protein, with average values of 5.2 and 7.1 Å, respectively. Notably, pyronaridine displayed a CoM distance of about 2.1 Å less than that of R6S complexed with the BRD4-BD1 protein throughout the simulation period. This may be attributed to the difference in the molecular weight of the two inhibitors. These steady distance variations proved that the investigated inhibitors could stably interact with the critical residues.

#### 2.4.4. Root-Mean-Square Deviation (RMSD)

**Figure 9:**
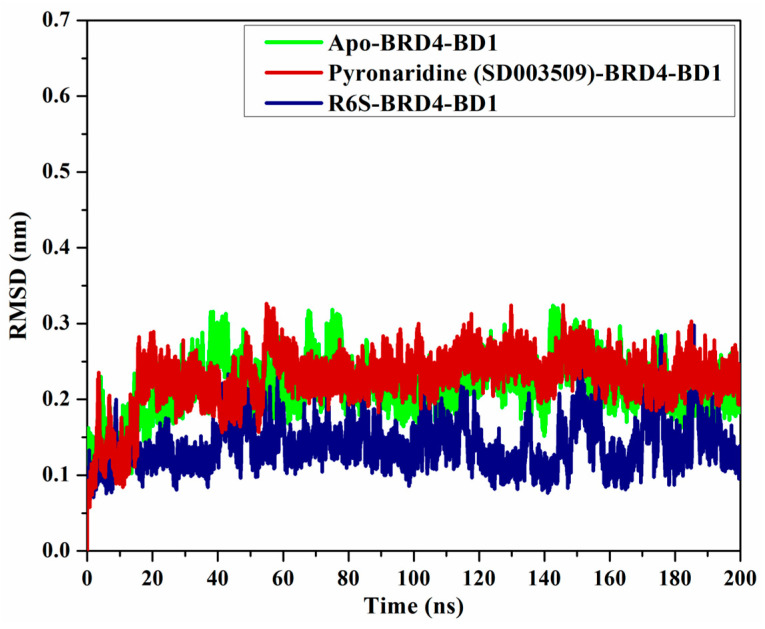
RMSD of apo-BRD4-BD1 (in green), pyronaridine (SD003509)-BRD4-BD1 (in dark red), and R6S-BRD4-BD1 (in navy) throughout the MD simulation.

To check the structural variations of BRD4-BD1 complexed with pyronaridine and R6S, the RMSD of the backbone atoms, with respect to the first frame, was measured (Figure 9). The apo-, pyronaridine-, and R6S-BRD4-BD1 complexes exhibited overall steadiness, with average RMSD values of 0.22, 0.22, and 0.14 nm, respectively. A slight difference of 0.08 nm was observed between the RMSD curves of the pyronaridine- and R6S-BRD4-BD1 complexes, demonstrating the similar dynamics of the two systems. According to the current findings, pyronaridine does not disturb the overall structure of the BRD4-BD1 protein.

#### 2.4.5. Root-Mean-Square Fluctuation (RMSF)

**Figure 10:**
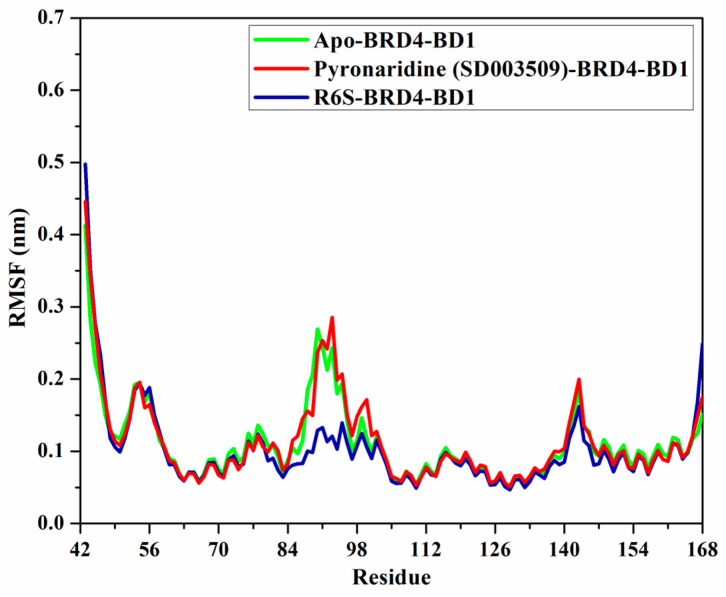
RMSF of apo-BRD4-BD1 (in green), pyronaridine (SD003509)-BRD4-BD1 (in dark red), and R6S-BRD4-BD1 (in navy) throughout the 200 ns MD simulations.

The RMSF of the C*_α_* atoms was studied to investigate the backbone conformational variation and stability of the apo-, pyronaridine-, and R6S-BRD4-BD1 complexes (Figure 10). As shown in Figure 10, the average RMSF values were 0.11, 0.11, and 0.10 nm for the apo-, pyronaridine-, and R6S-BRD4-BD1 complexes, respectively. In addition, the RMSF values of residues 84 to 100 in the R6S-BRD4-BD1 complex were lower than those of apo- and pyronaridine-BRD4-BD1, demonstrating that R6S tends to reduce this region’s flexibility. The larger size of the R6S inhibitor may be the reason for this rigidity. Overall, the RMSF results revealed that the amino acid residues were stationary during the 200 ns MD simulations in the identified compound complex, which is in line with the RMSD data.

### 2.5. ADMET Characteristics

Particularly in the precocious stages of medication discovery, investigating ADMET characteristics is a convenient tool for overcoming the limitations of clinical trials [34]. Consequently, the pkCSM webserver was applied to study the pharmacokinetic features of pyronaridine and R6S as BRD4-BD1 inhibitors. The pharmacokinetic features were predicted and gathered in Table 3. In all drug discovery processes, it is necessary to consider absorption features such as Caco-2 permeability and HIA. The absorbance rates of pyronaridine (93.6%) and R6S (90.8%) were favorable [35]. Both pyronaridine and R6S demonstrated low Caco-2 permeability (<0.9 cm/s). The VDss characteristics were evaluated to investigate the drug distribution [36]. Pyronaridine and R6S had high distribution volumes, with log VDss values of 1.41 and 1.15, respectively. The total clearance of the investigated inhibitors was calculated by multiplying their excretion rates by their concentrations in the body (Table 3). The excretion rates were 0.5 and 1.2 mL/min/kg for pyronaridine and R6S, respectively. Furthermore, toxicity is crucial to identifying suitable therapeutic compounds [36]. Pyronaridine and R6S do not have any AMES toxicity.

### 2.6. Drug-Likeness Characteristics

Using the Molinspiration online tool, physicochemical properties were predicted for pyronaridine and R6S and are listed in Table 4. As shown in Table 4, the Mlog*P* value of pyronaridine was below five (calc. 2.8), indicating that pyronaridine has a good permeability across the cell membrane. However, the Mlog*P* value of R6S was above five (calc. 6.0). The pyronaridine and R6S were predicted to be easily transported, dispersed, and absorbed since their molecular weight was found to be slightly more than 500 (calc. 520.1 and 552.7 dalton, respectively). Additionally, consistent with Lipinski’s rule of five (RO5), the number of hydrogen bond acceptors (nON) and donors (nOHNH) of the two compounds were less than 10 and 5, respectively. Furthermore, the TPSA values of the two compounds were found to be less than 140 Å^2^ (calc. 76.1 and 90.5 Å^2^, respectively). The two compounds may have potent cell membrane permeability and oral bioavailability, as shown by the calculated %ABS data (calc. 82.73% and 77.79%, respectively).

## 3. Computational Methods

### 3.1. BRD4-BD1 Preparation

The 3D structure of BRD4-BD1 bound to R6S (PDB code: 7REK [25]) was obtained and prepared for *in silico* analysis. For preparation purposes, crystallographic water molecules, R6S, and ions were eliminated. The H++ web-based server was utilized for the investigation of the protonation states of the BRD4-BD1 residues [37].

### 3.2. Database Preparation

The SuperDRUG2 database (http://cheminfo.charite.de/superdrug2 (accessed on 9 June 2023)), which includes more than 4600 candidates, was accessed in SDF format [38]. On the basis of InChIKey (International Chemical Identifier), the duplicated compounds were eliminated. The Omega 2.5.1.4 software was then utilized for the generation of the three-dimensional chemical structures of the inhibitors [39,40]. Using the SZYBKI 1.9.0.3 software, the three-dimensional structures were minimized based on MMFF94S force fields [41,42]. The prepared SuperDRUG2 database was applicable through www.compchem.net/ccdb (accessed on 9 June 2023).

### 3.3. Docking Calculations

All docking computations were executed utilizing the AutoDock Vina1.1.2 software [43]. The BRD4-BD1 pdbqt file was prepared for molecular docking using MGL tools (version 1.5.7) [44]. Two stages of docking calculations were performed, namely, standard and expensive docking computations. All docking parameters, except the exhaustiveness value, were set to their default parameters. For standard and expensive computations, the exhaustiveness values were modified to 50 and 200, respectively. The grid size was designed to have XYZ dimensions of 15 Å × 15 Å × 15 Å, encompassing the binding pocket. The grid spacing value was 1.0 Å. Additionally, the grid was centered at 6.636, 9.814, and −49.078 (in XYZ coordinates) for the BRD4-BD1 protein.

### 3.4. Molecular Dynamics (MD)

MD simulations were accomplished utilizing the AMBER16 software [45]. MD simulation details are described elsewhere [46,47,48]. In summary, BRD4-BD1 was characterized employing the FF14SB AMBER force field, whereas the inhibitors were parameterized through GAFF2 (the general AMBER force field) [49,50]. The charges of the studied drugs were determined using the RESP (restrained electrostatic potential) approach. Prior to charge estimations, the investigated compounds were optimized at the HF/6-31G* level of theory using the Gaussian09 software [51,52]. Inhibitor-BRD4-BD1 complexes were solvated in a truncated octahedron water box with a 12 Å distance. To neutralize the solvated systems and preserve the isosmotic condition, Na^+^ and Cl^−^ were added. To eliminate geometrical clashes, the constructed complexes were first minimized for 5000 cycles. Thereafter, the complexes were slowly warmed up for 50 ps up to 310 K. After that, a production step lasting 200 ns was carried out after an equilibration stage of 10 ns. The frames were collected every 10 ps for post-dynamic analyses. All MD simulations were conducted using the CompChem GPU/CPU hybrid cluster (hpc.compchem.net (accessed on 9 June 2023)). All visualizations were implemented utilizing BIOVIA DS Visualize 2020 [53].

### 3.5. Binding Energy Evaluation

The binding energy of the investigated inhibitors complexed with BRD4-BD1 was computed utilizing MM-GBSA (molecular mechanics–generalized Born surface area) approach based on the gathered snapshots [54]. The following equation was utilized to determine the MM-GBSA binding energy:

∆*G*_binding_ = *G*_Complex_ − (*G*_drugs_ + *G*_BRD4-BD1_) (1)

The *G* term was evaluated as

*G* = *G*_SA_ + *E*_vdw_ + *G*_GB_ + *E*_ele_ (2)

*E*_ele_ indicates electrostatic energy. *G*_SA_ stands for nonpolar solvation-free energy. *E*_vdW_ is van der Waals energy. *G*_GB_ is the electrostatic solvation energy. The entropy of the inhibitors complexed with BRD4-BD1 was ignored because of its high computing expense [55,56].

### 3.6. ADMET Characteristics

The ADMET (absorption, distribution, metabolism, excretion, and toxicity) values of the investigated inhibitors were anticipated using pkCSM (http://biosig.unimelb.edu.au/pkcsm/prediction (accessed on 9 June 2023)) [34]. The absorption characteristic involved Caco-2 permeability and HIA (human intestinal absorption). The distribution was anticipated using the VDss (steady-state volume of distribution). The metabolism was estimated based on the CYP3A4 substrate/inhibitor. The excretion was determined via the drug total clearance. Based on AMES toxicity, the toxicity was predicted [57].

### 3.7. Drug-Likeness Characteristics

The physicochemical properties of the identified BRD4-BD1 drug candidate were calculated using the online cheminformatics tool Molinspiration (http://www.molinspiration.com (accessed on 9 June 2023)). The topological polar surface area (TPSA), the number of hydrogen bond donors (nOHNH), the number of hydrogen bond acceptors (nON), the molecular weight (MWt), the percentage of absorption (%ABS), and the partition coefficient log P (MLog*P*) parameters were assessed. The %ABS was evaluated as follows [58]:

%ABS = 109 − [0.345 × TPSA]

## 4. Conclusions

*N*-terminal bromodomain (BD1) is essential for disrupting BRD4 interactions and is a promising protein for cancer therapy. The SuperDRUG2 database, containing over 4600 pharmaceutical compounds, was virtually screened against the BRD4-BD1 binding pocket utilizing molecular docking and MD simulations. The binding affinities of the identified inhibitors were then evaluated utilizing the MM-GBSA approach. According to the presented results, only three inhibitors out of the SuperDRUG2 database disclosed docking smaller scores than those of the R6S inhibitor (calc. −9.9 kcal/mol) within the BRD4-BD1 binding pocket. Interestingly, over a simulation time of 200 ns, pyronaridine (SD003509) revealed a binding affinity with a ∆*G*_binding_ value of −42.7 kcal/mol against BRD4-BD1, which was less than that of R6S (calc. −41.5 kcal/mol). Furthermore, energetical and structural analyses revealed the steadiness of the pyronaridine- and R6S-RD4-BD1 complexes during a simulation course of 200 ns. It is worth mentioning that *E*_vdW_ and *E*_ele_ considerably contributed to pyronaridine- and R6S-BRD4-BD1 binding affinities. Additionally, the physicochemical and ADMET properties of pyronaridine were promising. Experimental investigations are planned to explain the function of pyronaridine as a potential anticancer medication.

## Figures and Tables

**Table 1:** Evaluated docking scores and binding features of the top three inhibitors with BRD4-BD1 ^a^.

No.	Inhibitor Name/Code	Two-Dimensional Chemical Structure	Docking Score (kcal/mol)		Binding Features
			Standard	Expensive	
	R6S	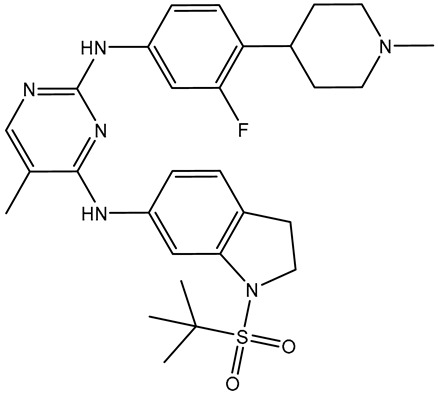	−9.9	−10.0	ASN140 (H-bond, 2.71, 1.92 Å), PRO82 (H-bond, 3.04 Å; π-Alkyl, 5.07 Å), LYS91 (H- bond, 2.45 Å), ASP88 (H- bond, 1.93 Å), LEU92 (π-Alkyl, 4.75, 4.85, 5.49 Å), VAL87 (π-Alkyl, 5.22, 4.92 Å), LEU94 (π-Alkyl, 5.10 Å), PHE83 (π-Alkyl, 4.17 Å), CYS136 (π-Alkyl, 5.36 Å), ILE146 (π-Alkyl, 4.19 Å), TRP81 (π-Alkyl, 5.03 Å; π-π T-shaped, 5.25 Å)
1	Pyronaridine (SD003509)	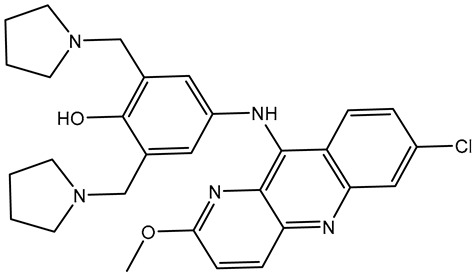	−10.1	−10.2	ASN140 (H-bond, 2.32, 2.06 Å), TYR97 (H-bond, 2.67 Å), ASP88 (H-bond, 1.95 Å), PRO82 (H-bond, 2.23 Å; π-Alkyl, 5.13, 5.47 Å), LEU92 (π-Alkyl, 4.39, 4.68 Å), VAL87 (π-Alkyl, 4.44 Å), ILE146 (π-Alkyl, 4.90 Å), TRRP81 (π-π T-shaped, 4.89, 4.92 Å)
2	Lumacaftor (SD003873)	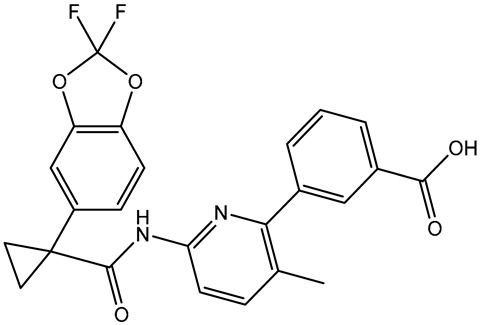	−10.1	−10.1	GLN85 (H-bond, 2.45, 3.08 Å), LEU92 (π-Alkyl, 4.42 Å), VAL87 (π-Alkyl, 4.87 Å), ILE146 (π-Alkyl, 4.21, 4.68 Å), TRP81 (π-Alkyl, 4.87 Å; π-π T-shaped, 5.15 Å)
3	*N*-benzoylstaurosporine (SD006001)	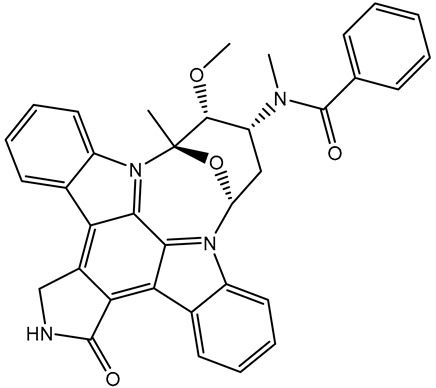	−9.9	−10.0	LEU92 (π-Alkyl, 4.50, 4.99 Å), VAL87 (π-Alkyl, 5.22, 4.86 Å), CYS136 (π-Alkyl, 5.13 Å), TRRP81 (π-π T-shaped, 5.29, 5.21 Å)

^a^ Data sorted in accordance with the expensive docking scores.

**Table 2:** Calculated binding energies (in kcal/mol) throughout the 50 and 200 ns MD simulations for the three most potential drug candidates complexed with the BRD4-BD1 protein.

Inhibitor Name/SuperDRUG2 Code	MM-GBSA Binding Energy (kcal/mol)	
	50 ns	200 ns
R6S	−43.9	−41.5
Pyronaridine (SD003509)	−46.2	−42.7
Lumacaftor (SD003873)	−27.8	--- ^a^
*N*-benzoylstaurosporine (SD006001)	−20.0	--- ^a^

^a^ Not calculated.

**Table 3:** Predicted ADMET properties of the identified drug candidate compared with R6S utilizing the pkCSM server.

Inhibitor Code	Absorption (A)		Distribution (D)	Metabolism (M)	Excretion (E)	Toxicity (T)
	Caco2 Permeability (cm/s)	Human Intestinal Absorption (HIA)	VDss (Human)	CYP3A4 Inhibitor/Substrate	Total Clearance	AMES Toxicity
Pyronaridine (SD003509)	0.62	93.60	1.41	Yes	1.21	No
R6S	1.12	90.84	1.15	Yes	0.57	No

**Table 4:** The investigated physicochemical properties of the identified drug candidate and R6S.

Compound Name	MLog*P*	TPSA	nON	nOHNH	Nrotb	MWt	%ABS
R6S	6.0	76.1	7	2	6	552.7	82.7
Pyronaridine (SD003509)	2.8	90.5	5	4	7	520.1	77.8

## Data Availability

The data presented in this study are available in the Supplementary Material.

## Supplementary Materials

The following supporting information can be downloaded at https://www.mdpi.com/article/10.3390/molecules28155713/s1: Table S1. Evaluated standard docking scores (in kcal/mol) for the investigated compounds with docking scores less than −7.0 kcal/mol against the BRD4-BD1 (exhaustiveness number = 50).
