# Supplementary material for: Pyronaridine as a Bromodomain-Containing Protein 4-N-Terminal Bromodomain (BRD4-BD1) Inhibitor: In Silico Database Mining, Molecular Docking, and Molecular Dynamics Simulation
Source: Molecules. 2023 Jul 28;28(15):5713. doi: 10.3390/molecules28155713 (PMC10420099; doi:10.3390/molecules28155713)
Supplement: Supplementary file 1 [file molecules-28-05713-s001.zip › molecules-2471499-supplementary.pdf]

Table S1. Evaluated standard docking scores (in kcal/mol) for the investigated compounds with docking scores less than –7.0 kcal/mol against the BRD4-BD1 (Exhaustiveness number = 50).

No.	SuperDRUG2 Code	Standard Docking Score (kcal/mol)	No.	SuperDRUG2 Code	Standard Docking Score (kcal/mol)	No.	SuperDRUG2 Code	Standard Docking Score (kcal/mol)
	**R6S**	**–9.9**	48	SD003836	–9.1	96	SD000605	–8.9
1	SD003509	–10.1	49	SD006034	–9.1	97	SD000929	–8.8
2	SD003873	–10.1	50	SD000645	–9.1	98	SD000896	–8.8
3	SD006001	–9.9	51	SD000946	–9.0	99	SD000863	–8.8
4	SD003842	–9.8	52	SD001084	–9.0	100	SD001074	–8.8
5	SD000497	–9.8	53	SD003217	–9.0	101	SD001051	–8.8
6	SD001949	–9.7	54	SD001678	–9.0	102	SD001271	–8.8
7	SD003490	–9.7	55	SD000012	–9.0	103	SD001279	–8.8
8	SD000062	–9.7	56	SD001987	–9.0	104	SD001274	–8.8
9	SD000944	–9.6	57	SD002471	–9.0	105	SD000140	–8.8
10	SD001524	–9.6	58	SD000265	–9.0	106	SD000146	–8.8
11	SD002150	–9.6	59	SD000306	–9.0	107	SD001638	–8.8
12	SD002280	–9.6	60	SD000330	–9.0	108	SD001367	–8.8
13	SD003376	–9.6	61	SD002620	–9.0	109	SD001865	–8.8
14	SD003145	–9.6	62	SD003573	–9.0	110	SD001599	–8.8
15	SD003291	–9.5	63	SD003749	–9.0	111	SD002051	–8.8
16	SD002790	–9.5	64	SD003802	–9.0	112	SD002114	–8.8
17	SD000943	–9.4	65	SD003939	–9.0	113	SD002115	–8.8
18	SD001942	–9.4	66	SD000412	–9.0	114	SD002322	–8.8
19	SD002303	–9.4	67	SD000414	–9.0	115	SD002506	–8.8
20	SD002172	–9.4	68	SD000719	–9.0	116	SD002417	–8.8
21	SD002496	–9.4	69	SD000709	–9.0	117	SD002582	–8.8
22	SD000686	–9.4	70	SD000839	–9.0	118	SD002800	–8.8
23	SD000883	–9.3	71	SD000858	–8.9	119	SD003415	–8.8
24	SD003224	–9.3	72	SD001004	–8.9	120	SD003151	–8.8
25	SD003820	–9.3	73	SD001347	–8.9	121	SD003543	–8.8
26	SD003943	–9.3	74	SD001419	–8.9	122	SD003714	–8.8
27	SD006014	–9.3	75	SD001471	–8.9	123	SD003791	–8.8
28	SD001640	–9.2	76	SD001406	–8.9	124	SD003876	–8.8
29	SD003076	–9.2	77	SD001705	–8.9	125	SD003905	–8.8
30	SD002297	–9.2	78	SD001634	–8.9	126	SD003930	–8.8
31	SD003363	–9.2	79	SD000168	–8.9	127	SD000575	–8.8
32	SD002572	–9.2	80	SD001727	–8.9	128	SD000691	–8.8
33	SD003570	–9.2	81	SD001623	–8.9	129	SD000671	–8.8
34	SD003940	–9.2	82	SD001776	–8.9	130	SD000735	–8.8
35	SD000554	–9.2	83	SD000199	–8.9	131	SD000895	–8.7
36	SD000846	–9.2	84	SD002006	–8.9	132	SD000949	–8.7
37	SD001057	–9.1	85	SD003341	–8.9	133	SD001025	–8.7
38	SD001459	–9.1	86	SD002373	–8.9	134	SD001468	–8.7
39	SD001505	–9.1	87	SD002633	–8.9	135	SD001854	–8.7
40	SD001644	–9.1	88	SD002712	–8.9	136	SD001910	–8.7
41	SD001676	–9.1	89	SD002713	–8.9	137	SD002027	–8.7
42	SD001713	–9.1	90	SD003034	–8.9	138	SD002123	–8.7
43	SD001824	–9.1	91	SD003738	–8.9	139	SD002218	–8.7
44	SD002018	–9.1	92	SD003901	–8.9	140	SD002702	–8.7
45	SD003347	–9.1	93	SD003895	–8.9	141	SD003385	–8.7
46	SD002327	–9.1	94	SD006006	–8.9	142	SD003400	–8.7
47	SD003350	–9.1	95	SD006007	–8.9	143	SD003127	–8.7

Table S1. *Continued.*

No.	SuperDRUG2 Code	Standard Docking Score (kcal/mol)	No.	SuperDRUG2 Code	Standard Docking Score (kcal/mol)	No.	SuperDRUG2 Code	Standard Docking Score (kcal/mol)
144	SD003706	–8.7	192	SD001104	–8.5	240	SD000188	–8.4
145	SD003764	–8.7	193	SD001160	–8.5	241	SD001915	–8.4
146	SD003908	–8.7	194	SD001156	–8.5	242	SD001954	–8.4
147	SD006052	–8.7	195	SD001534	–8.5	243	SD001982	–8.4
148	SD003937	–8.7	196	SD001481	–8.5	244	SD002024	–8.4
149	SD000438	–8.7	197	SD001564	–8.5	245	SD001969	–8.4
150	SD000495	–8.7	198	SD001602	–8.5	246	SD002106	–8.4
151	SD000606	–8.7	199	SD001688	–8.5	247	SD002007	–8.4
152	SD000674	–8.7	200	SD001617	–8.5	248	SD000206	–8.4
153	SD000725	–8.7	201	SD001858	–8.5	249	SD003306	–8.4
154	SD000928	–8.6	202	SD000192	–8.5	250	SD002182	–8.4
155	SD000952	–8.6	203	SD002098	–8.5	251	SD002207	–8.4
156	SD001048	–8.6	204	SD002133	–8.5	252	SD002145	–8.4
157	SD001107	–8.6	205	SD002120	–8.5	253	SD000222	–8.4
158	SD000145	–8.6	206	SD002129	–8.5	254	SD002277	–8.4
159	SD001575	–8.6	207	SD002200	–8.5	255	SD002287	–8.4
160	SD001551	–8.6	208	SD002255	–8.5	256	SD003353	–8.4
161	SD000176	–8.6	209	SD003337	–8.5	257	SD002559	–8.4
162	SD001849	–8.6	210	SD002345	–8.5	258	SD002593	–8.4
163	SD003273	–8.6	211	SD002441	–8.5	259	SD002740	–8.4
164	SD002045	–8.6	212	SD002510	–8.5	260	SD003407	–8.4
165	SD002108	–8.6	213	SD000261	–8.5	261	SD002708	–8.4
166	SD002192	–8.6	214	SD002760	–8.5	262	SD002534	–8.4
167	SD002225	–8.6	215	SD003417	–8.5	263	SD003413	–8.4
168	SD000225	–8.6	216	SD003044	–8.5	264	SD003439	–8.4
169	SD002323	–8.6	217	SD003545	–8.5	265	SD003093	–8.4
170	SD002845	–8.6	218	SD003515	–8.5	266	SD000308	–8.4
171	SD003412	–8.6	219	SD000351	–8.5	267	SD000315	–8.4
172	SD003052	–8.6	220	SD003778	–8.5	268	SD003531	–8.4
173	SD003103	–8.6	221	SD003857	–8.5	269	SD003596	–8.4
174	SD003487	–8.6	222	SD003878	–8.5	270	SD003622	–8.4
175	SD003497	–8.6	223	SD006047	–8.5	271	SD003624	–8.4
176	SD003643	–8.6	224	SD003711	–8.5	272	SD003716	–8.4
177	SD003818	–8.6	225	SD000574	–8.5	273	SD003774	–8.4
178	SD003828	–8.6	226	SD000724	–8.5	274	SD003813	–8.4
179	SD003854	–8.6	227	SD000968	–8.4	275	SD003767	–8.4
180	SD006050	–8.6	228	SD003175	–8.4	276	SD003941	–8.4
181	SD000447	–8.6	229	SD000983	–8.4	277	SD006036	–8.4
182	SD000660	–8.6	230	SD001068	–8.4	278	SD000057	–8.4
183	SD000700	–8.6	231	SD001095	–8.4	280	SD006013	–8.4
184	SD000726	–8.6	232	SD000119	–8.4	281	SD000404	–8.4
185	SD000874	–8.5	233	SD001313	–8.4	282	SD000407	–8.4
186	SD000855	–8.5	234	SD001489	–8.4	283	SD000448	–8.4
187	SD000856	–8.5	235	SD003242	–8.4	279	SD000702	–8.4
188	SD001002	–8.5	236	SD000158	–8.4	284	SD000699	–8.4
189	SD001015	–8.5	237	SD003264	–8.4	285	SD000816	–8.4
190	SD001033	–8.5	238	SD001683	–8.4	286	SD000782	–8.4
191	SD000105	–8.5	239	SD001802	–8.4	287	SD000787	–8.4

Table S1. *Continued.*

No.	SuperDRUG2 Code	Standard Docking Score (kcal/mol)	No.	SuperDRUG2 Code	Standard Docking Score (kcal/mol)	No.	SuperDRUG2 Code	Standard Docking Score (kcal/mol)
288	SD000867	–8.3	336	SD006011	–8.3	384	SD002179	–8.2
289	SD000966	–8.3	337	SD000076	–8.3	385	SD001668	–8.2
290	SD000994	–8.3	338	SD000410	–8.3	386	SD002188	–8.2
291	SD001052	–8.3	339	SD000413	–8.3	387	SD002269	–8.2
292	SD003180	–8.3	340	SD000564	–8.3	388	SD002190	–8.2
293	SD001001	–8.3	341	SD000567	–8.3	389	SD002305	–8.2
294	SD001158	–8.3	342	SD000662	–8.3	390	SD003362	–8.2
295	SD001228	–8.3	343	SD000703	–8.3	391	SD002460	–8.2
296	SD001153	–8.3	344	SD000783	–8.3	392	SD002448	–8.2
297	SD001157	–8.3	345	SD000790	–8.3	393	SD002470	–8.2
298	SD001445	–8.3	346	SD000848	–8.2	394	SD002575	–8.2
299	SD001467	–8.3	347	SD000097	–8.2	395	SD002561	–8.2
300	SD003226	–8.3	348	SD001100	–8.2	396	SD003382	–8.2
301	SD001578	–8.3	349	SD000091	–8.2	397	SD002733	–8.2
302	SD001580	–8.3	350	SD001161	–8.2	398	SD003405	–8.2
303	SD001571	–8.3	351	SD001154	–8.2	399	SD002795	–8.2
304	SD003262	–8.3	352	SD001236	–8.2	400	SD002773	–8.2
305	SD001807	–8.3	353	SD001319	–8.2	401	SD002782	–8.2
306	SD001852	–8.3	354	SD000127	–8.2	402	SD002895	–8.2
307	SD001818	–8.3	355	SD001409	–8.2	403	SD002932	–8.2
308	SD001885	–8.3	356	SD001412	–8.2	404	SD003419	–8.2
309	SD001893	–8.3	357	SD001374	–8.2	405	SD003016	–8.2
310	SD001939	–8.3	358	SD001416	–8.2	406	SD003438	–8.2
311	SD002088	–8.3	359	SD001447	–8.2	407	SD003132	–8.2
312	SD002105	–8.3	360	SD001278	–8.2	408	SD000312	–8.2
313	SD002186	–8.3	361	SD001515	–8.2	409	SD003505	–8.2
314	SD000214	–8.3	362	SD001604	–8.2	410	SD003488	–8.2
315	SD002189	–8.3	363	SD001675	–8.2	411	SD000328	–8.2
316	SD002170	–8.3	364	SD001711	–8.2	412	SD003615	–8.2
317	SD002459	–8.3	365	SD001658	–8.2	413	SD003701	–8.2
318	SD002436	–8.3	366	SD001635	–8.2	414	SD003688	–8.2
319	SD002524	–8.3	367	SD001762	–8.2	415	SD003776	–8.2
320	SD000249	–8.3	368	SD001111	–8.2	416	SD000361	–8.2
321	SD002734	–8.3	369	SD001763	–8.2	417	SD003832	–8.2
322	SD002850	–8.3	370	SD001820	–8.2	418	SD003883	–8.2
323	SD003020	–8.3	371	SD001859	–8.2	419	SD003945	–8.2
324	SD002963	–8.3	372	SD001889	–8.2	420	SD000068	–8.2
325	SD000320	–8.3	373	SD001909	–8.2	421	SD000386	–8.2
326	SD000326	–8.3	374	SD001933	–8.2	422	SD000582	–8.2
327	SD003547	–8.3	375	SD001965	–8.2	423	SD000609	–8.2
328	SD003549	–8.3	376	SD002014	–8.2	424	SD000652	–8.2
329	SD003703	–8.3	377	SD003303	–8.2	425	SD000781	–8.2
330	SD000358	–8.3	378	SD003301	–8.2	426	SD000840	–8.2
331	SD003814	–8.3	379	SD002082	–8.2	427	SD000796	–8.2
332	SD003796	–8.3	380	SD000208	–8.2	428	SD000945	–8.1
333	SD003835	–8.3	381	SD002157	–8.2	429	SD000989	–8.1
334	SD003900	–8.3	382	SD002116	–8.2	430	SD001044	–8.1
335	SD003769	–8.3	383	SD003320	–8.2	431	SD001054	–8.1

Table S1. *Continued.*

No.	SuperDRUG2 Code	Standard Docking Score (kcal/mol)	No.	SuperDRUG2 Code	Standard Docking Score (kcal/mol)	No.	SuperDRUG2 Code	Standard Docking Score (kcal/mol)
432	SD001090	–8.1	480	SD000273	–8.1	528	SD000151	–8.0
433	SD001159	–8.1	481	SD002826	–8.1	529	SD001366	–8.0
434	SD000942	–8.1	482	SD002779	–8.1	530	SD001680	–8.0
435	SD001170	–8.1	483	SD003434	–8.1	531	SD001689	–8.0
436	SD003184	–8.1	484	SD000307	–8.1	532	SD001682	–8.0
437	SD001200	–8.1	485	SD000310	–8.1	533	SD001712	–8.0
438	SD001260	–8.1	486	SD003387	–8.1	534	SD001756	–8.0
439	SD001335	–8.1	487	SD003581	–8.1	535	SD001783	–8.0
440	SD001395	–8.1	488	SD003631	–8.1	536	SD001751	–8.0
441	SD001420	–8.1	489	SD003626	–8.1	537	SD001822	–8.0
442	SD001512	–8.1	490	SD000352	–8.1	538	SD001946	–8.0
443	SD001516	–8.1	491	SD003772	–8.1	539	SD001955	–8.0
444	SD001584	–8.1	492	SD003719	–8.1	540	SD001872	–8.0
445	SD003243	–8.1	493	SD003720	–8.1	541	SD003289	–8.0
446	SD001179	–8.1	494	SD003794	–8.1	542	SD002022	–8.0
447	SD001706	–8.1	495	SD000359	–8.1	543	SD003309	–8.0
448	SD001754	–8.1	496	SD003862	–8.1	544	SD002009	–8.0
449	SD000178	–8.1	497	SD006027	–8.1	545	SD002084	–8.0
450	SD001801	–8.1	498	SD003931	–8.1	546	SD002117	–8.0
451	SD003270	–8.1	499	SD006051	–8.1	547	SD003325	–8.0
452	SD000190	–8.1	500	SD000546	–8.1	548	SD002208	–8.0
453	SD001766	–8.1	501	SD000623	–8.1	549	SD002274	–8.0
454	SD001937	–8.1	502	SD000470	–8.1	550	SD002254	–8.0
455	SD001961	–8.1	503	SD000471	–8.1	551	SD003346	–8.0
456	SD002010	–8.1	504	SD000710	–8.1	552	SD002278	–8.0
457	SD002019	–8.1	505	SD000770	–8.1	553	SD000226	–8.0
458	SD002015	–8.1	506	SD000870	–8.0	554	SD002298	–8.0
459	SD002053	–8.1	507	SD000865	–8.0	555	SD003329	–8.0
460	SD002058	–8.1	508	SD000991	–8.0	556	SD002421	–8.0
461	SD001831	–8.1	509	SD000996	–8.0	557	SD002476	–8.0
462	SD000210	–8.1	510	SD001036	–8.0	558	SD003355	–8.0
463	SD002195	–8.1	511	SD001043	–8.0	559	SD002681	–8.0
464	SD002271	–8.1	512	SD001046	–8.0	560	SD002586	–8.0
465	SD003345	–8.1	513	SD000948	–8.0	561	SD002690	–8.0
466	SD002289	–8.1	514	SD001080	–8.0	562	SD002718	–8.0
467	SD002339	–8.1	515	SD001102	–8.0	563	SD002698	–8.0
468	SD002388	–8.1	516	SD001143	–8.0	564	SD002699	–8.0
469	SD003354	–8.1	517	SD003191	–8.0	565	SD002759	–8.0
470	SD002438	–8.1	518	SD001162	–8.0	566	SD002859	–8.0
471	SD002440	–8.1	519	SD000112	–8.0	567	SD000278	–8.0
472	SD000241	–8.1	520	SD001155	–8.0	568	SD002875	–8.0
473	SD002560	–8.1	521	SD001235	–8.0	569	SD003026	–8.0
474	SD002570	–8.1	522	SD001263	–8.0	570	SD000294	–8.0
475	SD000248	–8.1	523	SD000125	–8.0	571	SD003436	–8.0
476	SD003049	–8.1	524	SD001387	–8.0	572	SD003040	–8.0
477	SD002626	–8.1	525	SD001421	–8.0	573	SD003032	–8.0
478	SD002696	–8.1	526	SD001474	–8.0	574	SD003481	–8.0
479	SD002700	–8.1	527	SD001476	–8.0	575	SD003575	–8.0

Table S1. *Continued.*

No.	SuperDRUG2 Code	Standard Docking Score (kcal/mol)	No.	SuperDRUG2 Code	Standard Docking Score (kcal/mol)	No.	SuperDRUG2 Code	Standard Docking Score (kcal/mol)
576	SD003712	–8.0	624	SD001899	–7.9	672	SD003734	–7.9
577	SD000362	–8.0	625	SD001773	–7.9	673	SD003754	–7.9
578	SD003834	–8.0	626	SD001936	–7.9	674	SD003830	–7.9
579	SD003866	–8.0	627	SD001884	–7.9	675	SD003833	–7.9
580	SD006017	–8.0	628	SD001775	–7.9	676	SD003847	–7.9
581	SD003947	–8.0	629	SD001948	–7.9	677	SD003879	–7.9
582	SD006053	–8.0	630	SD001994	–7.9	678	SD000367	–7.9
583	SD000385	–8.0	631	SD002028	–7.9	679	SD003924	–7.9
584	SD000408	–8.0	632	SD002004	–7.9	680	SD003928	–7.9
585	SD000423	–8.0	633	SD003075	–7.9	681	SD000034	–7.9
586	SD000544	–8.0	634	SD002109	–7.9	682	SD000523	–7.9
587	SD000646	–8.0	635	SD002125	–7.9	683	SD000591	–7.9
588	SD000774	–8.0	636	SD002128	–7.9	684	SD000628	–7.9
589	SD000701	–8.0	637	SD002292	–7.9	685	SD000618	–7.9
590	SD000813	–8.0	638	SD002283	–7.9	686	SD000624	–7.9
591	SD000789	–8.0	639	SD002279	–7.9	687	SD000647	–7.9
592	SD000687	–8.0	640	SD002383	–7.9	688	SD000572	–7.9
593	SD000884	–7.9	641	SD002406	–7.9	689	SD000844	–7.9
594	SD000885	–7.9	642	SD002369	–7.9	690	SD000845	–7.9
595	SD000913	–7.9	643	SD002428	–7.9	691	SD000785	–7.9
596	SD000860	–7.9	644	SD002371	–7.9	692	SD000737	–7.9
597	SD000004	–7.9	645	SD002462	–7.9	693	SD000815	–7.9
598	SD000879	–7.9	646	SD002477	–7.9	694	SD000688	–7.9
599	SD000977	–7.9	647	SD000020	–7.9	695	SD000894	–7.8
600	SD000993	–7.9	648	SD000256	–7.9	696	SD000005	–7.8
601	SD001061	–7.9	649	SD000257	–7.9	697	SD000975	–7.8
602	SD003179	–7.9	650	SD000260	–7.9	698	SD001039	–7.8
603	SD001077	–7.9	651	SD003398	–7.9	699	SD001041	–7.8
604	SD001085	–7.9	652	SD002723	–7.9	700	SD001022	–7.8
605	SD001079	–7.9	653	SD002533	–7.9	701	SD001093	–7.8
606	SD001067	–7.9	654	SD002817	–7.9	702	SD001075	–7.8
607	SD000926	–7.9	655	SD002846	–7.9	703	SD001047	–7.8
608	SD001196	–7.9	656	SD002786	–7.9	704	SD001126	–7.8
609	SD001353	–7.9	657	SD000280	–7.9	705	SD003171	–7.8
610	SD001345	–7.9	658	SD002872	–7.9	706	SD001149	–7.8
611	SD001413	–7.9	659	SD003422	–7.9	707	SD001244	–7.8
612	SD001434	–7.9	660	SD002948	–7.9	708	SD001262	–7.8
613	SD001422	–7.9	661	SD003425	–7.9	709	SD001322	–7.8
614	SD001510	–7.9	662	SD002537	–7.9	710	SD003064	–7.8
615	SD001568	–7.9	663	SD003031	–7.9	711	SD001381	–7.8
616	SD001453	–7.9	664	SD003449	–7.9	712	SD001392	–7.8
617	SD001655	–7.9	665	SD003149	–7.9	713	SD001411	–7.8
618	SD001686	–7.9	666	SD000025	–7.9	714	SD001426	–7.8
619	SD001690	–7.9	667	SD003559	–7.9	715	SD001446	–7.8
620	SD001757	–7.9	668	SD003582	–7.9	716	SD001415	–7.8
621	SD001110	–7.9	669	SD003585	–7.9	717	SD001424	–7.8
622	SD000184	–7.9	670	SD003654	–7.9	718	SD001450	–7.8
623	SD000189	–7.9	671	SD003537	–7.9	719	SD001469	–7.8

Table S1. *Continued.*

No.	SuperDRUG2 Code	Standard Docking Score (kcal/mol)	No.	SuperDRUG2 Code	Standard Docking Score (kcal/mol)	No.	SuperDRUG2 Code	Standard Docking Score (kcal/mol)
720	SD001533	–7.8	768	SD002939	–7.8	816	SD001150	–7.7
721	SD001536	–7.8	769	SD002999	–7.8	817	SD001089	–7.7
722	SD001539	–7.8	770	SD003429	–7.8	818	SD001227	–7.7
723	SD001596	–7.8	771	SD002964	–7.8	819	SD001253	–7.7
724	SD001628	–7.8	772	SD003039	–7.8	820	SD001251	–7.7
725	SD001572	–7.8	773	SD003143	–7.8	821	SD001272	–7.7
726	SD001685	–7.8	774	SD003125	–7.8	822	SD000126	–7.7
727	SD001677	–7.8	775	SD000317	–7.8	823	SD003210	–7.7
728	SD001720	–7.8	776	SD003571	–7.8	824	SD001308	–7.7
729	SD001753	–7.8	777	SD000349	–7.8	825	SD001451	–7.7
730	SD001755	–7.8	778	SD003658	–7.8	826	SD001276	–7.7
731	SD001803	–7.8	779	SD003534	–7.8	827	SD003239	–7.7
732	SD001796	–7.8	780	SD003740	–7.8	828	SD000147	–7.7
733	SD001797	–7.8	781	SD003729	–7.8	829	SD001543	–7.7
734	SD001800	–7.8	782	SD003869	–7.8	830	SD001569	–7.7
735	SD001888	–7.8	783	SD003874	–7.8	831	SD001611	–7.7
736	SD001938	–7.8	784	SD003858	–7.8	832	SD001694	–7.7
737	SD001873	–7.8	785	SD003888	–7.8	833	SD001726	–7.7
738	SD003300	–7.8	786	SD003942	–7.8	834	SD000932	–7.7
739	SD002020	–7.8	787	SD003916	–7.8	835	SD003260	–7.7
740	SD003308	–7.8	788	SD006048	–7.8	836	SD001709	–7.7
741	SD002016	–7.8	789	SD000396	–7.8	837	SD000174	–7.7
742	SD000205	–7.8	790	SD000379	–7.8	838	SD001778	–7.7
743	SD002165	–7.8	791	SD000406	–7.8	839	SD003265	–7.7
744	SD001833	–7.8	792	SD000532	–7.8	840	SD000177	–7.7
745	SD001666	–7.8	793	SD000562	–7.8	841	SD001782	–7.7
746	SD002201	–7.8	794	SD000576	–7.8	842	SD003277	–7.7
747	SD000221	–7.8	795	SD000633	–7.8	843	SD001825	–7.7
748	SD002203	–7.8	796	SD000635	–7.8	844	SD001847	–7.7
749	SD002346	–7.8	797	SD000651	–7.8	845	SD003193	–7.7
750	SD002387	–7.8	798	SD000657	–7.8	846	SD001867	–7.7
751	SD002324	–7.8	799	SD000723	–7.8	847	SD001901	–7.7
752	SD002370	–7.8	800	SD000508	–7.8	848	SD001883	–7.7
753	SD002447	–7.8	801	SD000914	–7.7	849	SD001931	–7.7
754	SD002492	–7.8	802	SD000974	–7.7	850	SD003295	–7.7
755	SD002522	–7.8	803	SD000985	–7.7	851	SD001894	–7.7
756	SD003360	–7.8	804	SD003177	–7.7	852	SD001977	–7.7
757	SD002504	–7.8	805	SD001042	–7.7	853	SD002011	–7.7
758	SD002630	–7.8	806	SD001070	–7.7	854	SD002013	–7.7
759	SD000024	–7.8	807	SD001021	–7.7	855	SD001600	–7.7
760	SD002636	–7.8	808	SD001092	–7.7	856	SD003307	–7.7
761	SD002695	–7.8	809	SD001072	–7.7	857	SD000204	–7.7
762	SD003364	–7.8	810	SD000924	–7.7	858	SD000198	–7.7
763	SD002714	–7.8	811	SD000090	–7.7	859	SD002044	–7.7
764	SD003401	–7.8	812	SD001066	–7.7	860	SD002075	–7.7
765	SD002918	–7.8	813	SD001119	–7.7	861	SD002110	–7.7
766	SD002883	–7.8	814	SD001049	–7.7	862	SD002119	–7.7
767	SD000287	–7.8	815	SD001121	–7.7	863	SD002134	–7.7

Table S1. *Continued.*

No.	SuperDRUG2 Code	Standard Docking Score (kcal/mol)	No.	SuperDRUG2 Code	Standard Docking Score (kcal/mol)	No.	SuperDRUG2 Code	Standard Docking Score (kcal/mol)
864	SD002191	–7.7	912	SD003781	–7.7	960	SD001549	–7.6
865	SD002136	–7.7	913	SD003870	–7.7	961	SD001483	–7.6
866	SD002217	–7.7	914	SD003863	–7.7	962	SD001645	–7.6
867	SD003340	–7.7	915	SD003889	–7.7	963	SD000161	–7.6
868	SD002313	–7.7	916	SD003917	–7.7	964	SD001719	–7.6
869	SD003358	–7.7	917	SD003946	–7.7	965	SD000171	–7.6
870	SD002307	–7.7	918	SD006022	–7.7	966	SD001760	–7.6
871	SD002411	–7.7	919	SD000036	–7.7	967	SD001112	–7.6
872	SD002439	–7.7	920	SD000390	–7.7	968	SD000179	–7.6
873	SD002457	–7.7	921	SD000397	–7.7	969	SD001750	–7.6
874	SD002488	–7.7	922	SD000402	–7.7	970	SD000186	–7.6
875	SD002486	–7.7	923	SD000518	–7.7	971	SD000182	–7.6
876	SD002501	–7.7	924	SD000599	–7.7	972	SD001921	–7.6
877	SD003378	–7.7	925	SD000626	–7.7	973	SD001767	–7.6
878	SD002531	–7.7	926	SD000629	–7.7	974	SD001844	–7.6
879	SD002562	–7.7	927	SD000617	–7.7	975	SD001957	–7.6
880	SD003384	–7.7	928	SD000480	–7.7	976	SD001993	–7.6
881	SD000262	–7.7	929	SD000675	–7.7	977	SD001981	–7.6
882	SD002707	–7.7	930	SD000706	–7.7	978	SD002021	–7.6
883	SD000266	–7.7	931	SD000803	–7.7	979	SD001988	–7.6
884	SD002756	–7.7	932	SD000820	–7.7	980	SD002029	–7.6
885	SD002758	–7.7	933	SD000794	–7.7	981	SD002012	–7.6
886	SD002749	–7.7	934	SD000791	–7.7	982	SD001975	–7.6
887	SD002763	–7.7	935	SD000087	–7.6	983	SD002080	–7.6
888	SD002767	–7.7	936	SD000893	–7.6	984	SD002089	–7.6
889	SD002772	–7.7	937	SD000877	–7.6	985	SD002141	–7.6
890	SD002796	–7.7	938	SD000083	–7.6	986	SD001667	–7.6
891	SD002755	–7.7	939	SD000922	–7.6	987	SD002253	–7.6
892	SD002834	–7.7	940	SD001006	–7.6	988	SD002357	–7.6
893	SD002829	–7.7	941	SD001029	–7.6	989	SD002341	–7.6
894	SD002802	–7.7	942	SD000102	–7.6	990	SD002473	–7.6
895	SD002866	–7.7	943	SD001078	–7.6	991	SD002505	–7.6
896	SD003420	–7.7	944	SD001108	–7.6	992	SD002425	–7.6
897	SD002959	–7.7	945	SD000941	–7.6	993	SD002547	–7.6
898	SD003414	–7.7	946	SD001205	–7.6	994	SD002517	–7.6
899	SD003041	–7.7	947	SD001233	–7.6	995	SD000245	–7.6
900	SD003051	–7.7	948	SD001217	–7.6	996	SD002567	–7.6
901	SD000314	–7.7	949	SD001234	–7.6	997	SD002530	–7.6
902	SD000316	–7.7	950	SD001219	–7.6	998	SD000254	–7.6
903	SD000319	–7.7	951	SD001328	–7.6	999	SD000021	–7.6
904	SD000331	–7.7	952	SD003067	–7.6	1000	SD002638	–7.6
905	SD000343	–7.7	953	SD001350	–7.6	1001	SD000264	–7.6
906	SD003601	–7.7	954	SD001418	–7.6	1002	SD002721	–7.6
907	SD003630	–7.7	955	SD001432	–7.6	1003	SD002705	–7.6
908	SD003665	–7.7	956	SD000144	–7.6	1004	SD002757	–7.6
909	SD003647	–7.7	957	SD001360	–7.6	1005	SD002741	–7.6
910	SD003726	–7.7	958	SD001506	–7.6	1006	SD002742	–7.6
911	SD003731	–7.7	959	SD001525	–7.6	1007	SD003402	–7.6

Table S1. *Continued.*

No.	SuperDRUG2 Code	Standard Docking Score (kcal/mol)	No.	SuperDRUG2 Code	Standard Docking Score (kcal/mol)	No.	SuperDRUG2 Code	Standard Docking Score (kcal/mol)
1008	SD002805	–7.6	1056	SD000670	–7.6	1104	SD003299	–7.5
1009	SD002898	–7.6	1057	SD000817	–7.6	1105	SD001966	–7.5
1010	SD002878	–7.6	1058	SD000809	–7.6	1106	SD002043	–7.5
1011	SD002865	–7.6	1059	SD000722	–7.6	1107	SD000197	–7.5
1012	SD002884	–7.6	1060	SD000889	–7.5	1108	SD002001	–7.5
1013	SD002991	–7.6	1061	SD000909	–7.5	1109	SD001997	–7.5
1014	SD002994	–7.6	1062	SD000872	–7.5	1110	SD003324	–7.5
1015	SD002962	–7.6	1063	SD000976	–7.5	1111	SD002196	–7.5
1016	SD003004	–7.6	1064	SD001030	–7.5	1112	SD002276	–7.5
1017	SD003029	–7.6	1065	SD000100	–7.5	1113	SD002295	–7.5
1018	SD003104	–7.6	1066	SD003182	–7.5	1114	SD002232	–7.5
1019	SD003134	–7.6	1067	SD001076	–7.5	1115	SD002352	–7.5
1020	SD000311	–7.6	1068	SD001026	–7.5	1116	SD002348	–7.5
1021	SD000321	–7.6	1069	SD000104	–7.5	1117	SD000231	–7.5
1022	SD003495	–7.6	1070	SD003178	–7.5	1118	SD002456	–7.5
1023	SD003510	–7.6	1071	SD001050	–7.5	1119	SD002223	–7.5
1024	SD003540	–7.6	1072	SD001125	–7.5	1120	SD002472	–7.5
1025	SD003603	–7.6	1073	SD001208	–7.5	1121	SD002475	–7.5
1026	SD003577	–7.6	1074	SD001226	–7.5	1122	SD002424	–7.5
1027	SD003704	–7.6	1075	SD001198	–7.5	1123	SD003366	–7.5
1028	SD003705	–7.6	1076	SD001339	–7.5	1124	SD000243	–7.5
1029	SD003724	–7.6	1077	SD001266	–7.5	1125	SD002502	–7.5
1030	SD003718	–7.6	1078	SD001370	–7.5	1126	SD002541	–7.5
1031	SD003744	–7.6	1079	SD001281	–7.5	1127	SD002684	–7.5
1032	SD003745	–7.6	1080	SD001282	–7.5	1128	SD002783	–7.5
1033	SD003739	–7.6	1081	SD001414	–7.5	1129	SD002753	–7.5
1034	SD003795	–7.6	1082	SD001464	–7.5	1130	SD002554	–7.5
1035	SD003821	–7.6	1083	SD003225	–7.5	1131	SD002798	–7.5
1036	SD003850	–7.6	1084	SD001503	–7.5	1132	SD000275	–7.5
1037	SD003829	–7.6	1085	SD000153	–7.5	1133	SD002780	–7.5
1038	SD003899	–7.6	1086	SD001570	–7.5	1134	SD002858	–7.5
1039	SD003865	–7.6	1087	SD001595	–7.5	1135	SD002860	–7.5
1040	SD003949	–7.6	1088	SD001679	–7.5	1136	SD003409	–7.5
1041	SD003904	–7.6	1089	SD001702	–7.5	1137	SD002903	–7.5
1042	SD006004	–7.6	1090	SD001181	–7.5	1138	SD002956	–7.5
1043	SD006030	–7.6	1091	SD001615	–7.5	1139	SD002960	–7.5
1044	SD006032	–7.6	1092	SD001805	–7.5	1140	SD000289	–7.5
1045	SD006015	–7.6	1093	SD001781	–7.5	1141	SD002990	–7.5
1046	SD000436	–7.6	1094	SD001813	–7.5	1142	SD003440	–7.5
1047	SD000550	–7.6	1095	SD001848	–7.5	1143	SD003142	–7.5
1048	SD000551	–7.6	1096	SD001868	–7.5	1144	SD003472	–7.5
1049	SD000555	–7.6	1097	SD001905	–7.5	1145	SD000327	–7.5
1050	SD000563	–7.6	1098	SD001923	–7.5	1146	SD003572	–7.5
1051	SD000580	–7.6	1099	SD001956	–7.5	1147	SD003602	–7.5
1052	SD000521	–7.6	1100	SD003282	–7.5	1148	SD003632	–7.5
1053	SD000620	–7.6	1101	SD001777	–7.5	1149	SD003514	–7.5
1054	SD000642	–7.6	1102	SD001962	–7.5	1150	SD003698	–7.5
1055	SD000615	–7.6	1103	SD001978	–7.5	1151	SD003687	–7.5

Table S1. *Continued.*

No.	SuperDRUG2 Code	Standard Docking Score (kcal/mol)	No.	SuperDRUG2 Code	Standard Docking Score (kcal/mol)	No.	SuperDRUG2 Code	Standard Docking Score (kcal/mol)
1152	SD003823	–7.5	1200	SD000169	–7.4	1248	SD003380	–7.4
1153	SD003826	–7.5	1201	SD001738	–7.4	1249	SD000267	–7.4
1154	SD003875	–7.5	1202	SD001779	–7.4	1250	SD002777	–7.4
1155	SD003766	–7.5	1203	SD000165	–7.4	1251	SD003411	–7.4
1156	SD003890	–7.5	1204	SD001823	–7.4	1252	SD000276	–7.4
1157	SD003907	–7.5	1205	SD001817	–7.4	1253	SD002801	–7.4
1158	SD000446	–7.5	1206	SD001841	–7.4	1254	SD002887	–7.4
1159	SD000589	–7.5	1207	SD001891	–7.4	1255	SD000281	–7.4
1160	SD000594	–7.5	1208	SD001929	–7.4	1256	SD002864	–7.4
1161	SD000704	–7.5	1209	SD001918	–7.4	1257	SD000284	–7.4
1162	SD000713	–7.5	1210	SD001843	–7.4	1258	SD002974	–7.4
1163	SD000714	–7.5	1211	SD002035	–7.4	1259	SD002993	–7.4
1164	SD000753	–7.5	1212	SD000193	–7.4	1260	SD003007	–7.4
1165	SD000658	–7.5	1213	SD002057	–7.4	1261	SD003091	–7.4
1166	SD000755	–7.5	1214	SD002063	–7.4	1262	SD003129	–7.4
1167	SD000717	–7.5	1215	SD003317	–7.4	1263	SD003057	–7.4
1168	SD000804	–7.5	1216	SD002130	–7.4	1264	SD003119	–7.4
1169	SD000509	–7.5	1217	SD002151	–7.4	1265	SD003121	–7.4
1170	SD000919	–7.4	1218	SD002118	–7.4	1266	SD003486	–7.4
1171	SD000859	–7.4	1219	SD002167	–7.4	1267	SD003482	–7.4
1172	SD000849	–7.4	1220	SD002168	–7.4	1268	SD000334	–7.4
1173	SD000905	–7.4	1221	SD002202	–7.4	1269	SD003530	–7.4
1174	SD000862	–7.4	1222	SD002312	–7.4	1270	SD000345	–7.4
1175	SD000961	–7.4	1223	SD002206	–7.4	1271	SD003595	–7.4
1176	SD001031	–7.4	1224	SD002336	–7.4	1272	SD003695	–7.4
1177	SD001032	–7.4	1225	SD002354	–7.4	1273	SD003809	–7.4
1178	SD001016	–7.4	1226	SD002304	–7.4	1274	SD003827	–7.4
1179	SD001059	–7.4	1227	SD002338	–7.4	1275	SD003885	–7.4
1180	SD000940	–7.4	1228	SD002318	–7.4	1276	SD006016	–7.4
1181	SD001225	–7.4	1229	SD002404	–7.4	1277	SD003927	–7.4
1182	SD001187	–7.4	1230	SD002325	–7.4	1278	SD006005	–7.4
1183	SD001222	–7.4	1231	SD000237	–7.4	1279	SD000054	–7.4
1184	SD001320	–7.4	1232	SD002458	–7.4	1280	SD000058	–7.4
1185	SD001352	–7.4	1233	SD002434	–7.4	1281	SD000070	–7.4
1186	SD001399	–7.4	1234	SD002490	–7.4	1282	SD000072	–7.4
1187	SD001425	–7.4	1235	SD002443	–7.4	1283	SD000049	–7.4
1188	SD001429	–7.4	1236	SD002381	–7.4	1284	SD000066	–7.4
1189	SD001431	–7.4	1237	SD002487	–7.4	1285	SD000400	–7.4
1190	SD003229	–7.4	1238	SD003367	–7.4	1286	SD000444	–7.4
1191	SD001531	–7.4	1239	SD002591	–7.4	1287	SD000477	–7.4
1192	SD001518	–7.4	1240	SD000251	–7.4	1288	SD000590	–7.4
1193	SD001508	–7.4	1241	SD002454	–7.4	1289	SD000603	–7.4
1194	SD001583	–7.4	1242	SD000255	–7.4	1290	SD000712	–7.4
1195	SD001597	–7.4	1243	SD002631	–7.4	1291	SD000672	–7.4
1196	SD001660	–7.4	1244	SD002585	–7.4	1292	SD000472	–7.4
1197	SD001699	–7.4	1245	SD003390	–7.4	1293	SD000752	–7.4
1198	SD001659	–7.4	1246	SD002419	–7.4	1294	SD000741	–7.4
1199	SD001710	–7.4	1247	SD002694	–7.4	1295	SD000802	–7.4

Table S1. *Continued.*

No.	SuperDRUG2 Code	Standard Docking Score (kcal/mol)	No.	SuperDRUG2 Code	Standard Docking Score (kcal/mol)	No.	SuperDRUG2 Code	Standard Docking Score (kcal/mol)
1296	SD000826	–7.4	1344	SD002103	–7.3	1392	SD003597	–7.3
1297	SD000797	–7.4	1345	SD002166	–7.3	1393	SD003621	–7.3
1298	SD000954	–7.3	1346	SD002146	–7.3	1394	SD003648	–7.3
1299	SD003059	–7.3	1347	SD002152	–7.3	1395	SD003568	–7.3
1300	SD000098	–7.3	1348	SD002242	–7.3	1396	SD003725	–7.3
1301	SD000997	–7.3	1349	SD002272	–7.3	1397	SD003690	–7.3
1302	SD000923	–7.3	1350	SD002197	–7.3	1398	SD003815	–7.3
1303	SD001018	–7.3	1351	SD002249	–7.3	1399	SD000373	–7.3
1304	SD001064	–7.3	1352	SD002286	–7.3	1400	SD006020	–7.3
1305	SD001055	–7.3	1353	SD000216	–7.3	1401	SD000044	–7.3
1306	SD001024	–7.3	1354	SD002363	–7.3	1402	SD000073	–7.3
1307	SD000950	–7.3	1355	SD002342	–7.3	1403	SD003936	–7.3
1308	SD001082	–7.3	1356	SD002365	–7.3	1404	SD000417	–7.3
1309	SD001083	–7.3	1357	SD002420	–7.3	1405	SD000437	–7.3
1310	SD001087	–7.3	1358	SD002429	–7.3	1406	SD000452	–7.3
1311	SD000111	–7.3	1359	SD002464	–7.3	1407	SD000476	–7.3
1312	SD001194	–7.3	1360	SD003079	–7.3	1408	SD000573	–7.3
1313	SD001105	–7.3	1361	SD002483	–7.3	1409	SD000720	–7.3
1314	SD003202	–7.3	1362	SD002565	–7.3	1410	SD000744	–7.3
1315	SD001218	–7.3	1363	SD002566	–7.3	1411	SD000747	–7.3
1316	SD003206	–7.3	1364	SD002574	–7.3	1412	SD000805	–7.3
1317	SD001214	–7.3	1365	SD002640	–7.3	1413	SD000847	–7.3
1318	SD003205	–7.3	1366	SD002643	–7.3	1414	SD000829	–7.3
1319	SD001391	–7.3	1367	SD000259	–7.3	1415	SD000811	–7.3
1320	SD001270	–7.3	1368	SD002682	–7.3	1416	SD000901	–7.2
1321	SD001372	–7.3	1369	SD002687	–7.3	1417	SD000921	–7.2
1322	SD001402	–7.3	1370	SD002628	–7.3	1418	SD000986	–7.2
1323	SD001472	–7.3	1371	SD002670	–7.3	1419	SD000987	–7.2
1324	SD001576	–7.3	1372	SD002808	–7.3	1420	SD001005	–7.2
1325	SD001619	–7.3	1373	SD003406	–7.3	1421	SD000095	–7.2
1326	SD001629	–7.3	1374	SD002934	–7.3	1422	SD000992	–7.2
1327	SD001647	–7.3	1375	SD002938	–7.3	1423	SD001040	–7.2
1328	SD001649	–7.3	1376	SD000288	–7.3	1424	SD001053	–7.2
1329	SD001703	–7.3	1377	SD002941	–7.3	1425	SD001065	–7.2
1330	SD001708	–7.3	1378	SD002949	–7.3	1426	SD001135	–7.2
1331	SD003259	–7.3	1379	SD003042	–7.3	1427	SD001088	–7.2
1332	SD000173	–7.3	1380	SD003133	–7.3	1428	SD000951	–7.2
1333	SD001739	–7.3	1381	SD000295	–7.3	1429	SD003194	–7.2
1334	SD003072	–7.3	1382	SD003379	–7.3	1430	SD001099	–7.2
1335	SD001788	–7.3	1383	SD003148	–7.3	1431	SD001183	–7.2
1336	SD001789	–7.3	1384	SD000305	–7.3	1432	SD001176	–7.2
1337	SD001862	–7.3	1385	SD003126	–7.3	1433	SD000124	–7.2
1338	SD001922	–7.3	1386	SD003479	–7.3	1434	SD001337	–7.2
1339	SD001925	–7.3	1387	SD003483	–7.3	1435	SD001371	–7.2
1340	SD002040	–7.3	1388	SD000322	–7.3	1436	SD001307	–7.2
1341	SD003272	–7.3	1389	SD000027	–7.3	1437	SD001437	–7.2
1342	SD003196	–7.3	1390	SD003592	–7.3	1438	SD001364	–7.2
1343	SD002008	–7.3	1391	SD003611	–7.3	1439	SD001277	–7.2

Table S1. *Continued.*

No.	SuperDRUG2 Code	Standard Docking Score (kcal/mol)	No.	SuperDRUG2 Code	Standard Docking Score (kcal/mol)	No.	SuperDRUG2 Code	Standard Docking Score (kcal/mol)
1440	SD001523	–7.2	1488	SD002920	–7.2	1536	SD000972	–7.1
1441	SD003238	–7.2	1489	SD002823	–7.2	1537	SD000864	–7.1
1442	SD001493	–7.2	1490	SD002971	–7.2	1538	SD001011	–7.1
1443	SD000155	–7.2	1491	SD002984	–7.2	1539	SD000984	–7.1
1444	SD001632	–7.2	1492	SD000293	–7.2	1540	SD000086	–7.1
1445	SD003245	–7.2	1493	SD003038	–7.2	1541	SD001037	–7.1
1446	SD001654	–7.2	1494	SD003046	–7.2	1542	SD000099	–7.1
1447	SD001669	–7.2	1495	SD000323	–7.2	1543	SD003176	–7.1
1448	SD001656	–7.2	1496	SD000030	–7.2	1544	SD001081	–7.1
1449	SD003256	–7.2	1497	SD003502	–7.2	1545	SD001123	–7.1
1450	SD001707	–7.2	1498	SD003562	–7.2	1546	SD003190	–7.1
1451	SD000164	–7.2	1499	SD003513	–7.2	1547	SD001171	–7.1
1452	SD001731	–7.2	1500	SD003583	–7.2	1548	SD001199	–7.1
1453	SD001748	–7.2	1501	SD000346	–7.2	1549	SD001188	–7.1
1454	SD001882	–7.2	1502	SD003590	–7.2	1550	SD001252	–7.1
1455	SD003074	–7.2	1503	SD003619	–7.2	1551	SD001250	–7.1
1456	SD001944	–7.2	1504	SD003676	–7.2	1552	SD001211	–7.1
1457	SD001952	–7.2	1505	SD003681	–7.2	1553	SD001362	–7.1
1458	SD003290	–7.2	1506	SD003732	–7.2	1554	SD001265	–7.1
1459	SD002081	–7.2	1507	SD003708	–7.2	1555	SD001348	–7.1
1460	SD002099	–7.2	1508	SD003535	–7.2	1556	SD001306	–7.1
1461	SD002124	–7.2	1509	SD003867	–7.2	1557	SD001309	–7.1
1462	SD002177	–7.2	1510	SD003838	–7.2	1558	SD001430	–7.1
1463	SD002113	–7.2	1511	SD003673	–7.2	1559	SD001435	–7.1
1464	SD002140	–7.2	1512	SD003768	–7.2	1560	SD001462	–7.1
1465	SD003304	–7.2	1513	SD006008	–7.2	1561	SD001443	–7.1
1466	SD002180	–7.2	1514	SD003909	–7.2	1562	SD001492	–7.1
1467	SD002154	–7.2	1515	SD000050	–7.2	1563	SD001557	–7.1
1468	SD002259	–7.2	1516	SD000061	–7.2	1564	SD001480	–7.1
1469	SD002335	–7.2	1517	SD000399	–7.2	1565	SD000152	–7.1
1470	SD002291	–7.2	1518	SD000405	–7.2	1566	SD001542	–7.1
1471	SD002407	–7.2	1519	SD000421	–7.2	1567	SD001639	–7.1
1472	SD002161	–7.2	1520	SD000549	–7.2	1568	SD001633	–7.1
1473	SD002433	–7.2	1521	SD000487	–7.2	1569	SD001695	–7.1
1474	SD002413	–7.2	1522	SD000595	–7.2	1570	SD001180	–7.1
1475	SD002538	–7.2	1523	SD000602	–7.2	1571	SD001722	–7.1
1476	SD000252	–7.2	1524	SD000636	–7.2	1572	SD001723	–7.1
1477	SD000258	–7.2	1525	SD000640	–7.2	1573	SD001681	–7.1
1478	SD002583	–7.2	1526	SD000762	–7.2	1574	SD000013	–7.1
1479	SD002693	–7.2	1527	SD000763	–7.2	1575	SD001749	–7.1
1480	SD002587	–7.2	1528	SD000768	–7.2	1576	SD001857	–7.1
1481	SD002724	–7.2	1529	SD000801	–7.2	1577	SD001765	–7.1
1482	SD002735	–7.2	1530	SD000837	–7.2	1578	SD001906	–7.1
1483	SD000268	–7.2	1531	SD000818	–7.2	1579	SD001916	–7.1
1484	SD002750	–7.2	1532	SD000842	–7.2	1580	SD001895	–7.1
1485	SD003408	–7.2	1533	SD000507	–7.2	1581	SD003297	–7.1
1486	SD002811	–7.2	1534	SD000002	–7.1	1582	SD003288	–7.1
1487	SD002788	–7.2	1535	SD000904	–7.1	1583	SD001986	–7.1

Table S1. *Continued.*

No.	SuperDRUG2 Code	Standard Docking Score (kcal/mol)	No.	SuperDRUG2 Code	Standard Docking Score (kcal/mol)	No.	SuperDRUG2 Code	Standard Docking Score (kcal/mol)
1584	SD003298	–7.1	1632	SD003773	–7.1	1680	SD001718	–7.0
1585	SD001984	–7.1	1633	SD003849	–7.1	1681	SD001730	–7.0
1586	SD002030	–7.1	1634	SD000376	–7.1	1682	SD001734	–7.0
1587	SD002042	–7.1	1635	SD003846	–7.1	1683	SD001809	–7.0
1588	SD001970	–7.1	1636	SD000067	–7.1	1684	SD002052	–7.0
1589	SD002156	–7.1	1637	SD000074	–7.1	1685	SD002055	–7.0
1590	SD000212	–7.1	1638	SD000392	–7.1	1686	SD002073	–7.0
1591	SD002226	–7.1	1639	SD000415	–7.1	1687	SD002095	–7.0
1592	SD002228	–7.1	1640	SD000552	–7.1	1688	SD002062	–7.0
1593	SD000219	–7.1	1641	SD000579	–7.1	1689	SD002096	–7.0
1594	SD002266	–7.1	1642	SD000637	–7.1	1690	SD002091	–7.0
1595	SD002245	–7.1	1643	SD000665	–7.1	1691	SD003314	–7.0
1596	SD002261	–7.1	1644	SD000571	–7.1	1692	SD002111	–7.0
1597	SD002246	–7.1	1645	SD000695	–7.1	1693	SD002181	–7.0
1598	SD002275	–7.1	1646	SD000656	–7.1	1694	SD000217	–7.0
1599	SD000228	–7.1	1647	SD000800	–7.1	1695	SD002268	–7.0
1600	SD002309	–7.1	1648	SD000771	–7.1	1696	SD002237	–7.0
1601	SD002224	–7.1	1649	SD000810	–7.1	1697	SD002284	–7.0
1602	SD002430	–7.1	1650	SD000886	–7.0	1698	SD002385	–7.0
1603	SD003359	–7.1	1651	SD000912	–7.0	1699	SD002347	–7.0
1604	SD002466	–7.1	1652	SD000903	–7.0	1700	SD003356	–7.0
1605	SD002445	–7.1	1653	SD000850	–7.0	1701	SD002455	–7.0
1606	SD002579	–7.1	1654	SD000888	–7.0	1702	SD002412	–7.0
1607	SD002452	–7.1	1655	SD003167	–7.0	1703	SD002427	–7.0
1608	SD002569	–7.1	1656	SD000907	–7.0	1704	SD002556	–7.0
1609	SD002540	–7.1	1657	SD000953	–7.0	1705	SD002539	–7.0
1610	SD003383	–7.1	1658	SD003161	–7.0	1706	SD002568	–7.0
1611	SD002551	–7.1	1659	SD000955	–7.0	1707	SD002521	–7.0
1612	SD002747	–7.1	1660	SD001101	–7.0	1708	SD002577	–7.0
1613	SD002748	–7.1	1661	SD001091	–7.0	1709	SD002507	–7.0
1614	SD000271	–7.1	1662	SD001191	–7.0	1710	SD002580	–7.0
1615	SD002809	–7.1	1663	SD001130	–7.0	1711	SD002689	–7.0
1616	SD002848	–7.1	1664	SD001184	–7.0	1712	SD002784	–7.0
1617	SD002815	–7.1	1665	SD001201	–7.0	1713	SD002746	–7.0
1618	SD002807	–7.1	1666	SD001273	–7.0	1714	SD002709	–7.0
1619	SD002912	–7.1	1667	SD001336	–7.0	1715	SD002825	–7.0
1620	SD002904	–7.1	1668	SD001355	–7.0	1716	SD002876	–7.0
1621	SD002957	–7.1	1669	SD000136	–7.0	1717	SD002901	–7.0
1622	SD002958	–7.1	1670	SD001403	–7.0	1718	SD000283	–7.0
1623	SD002946	–7.1	1671	SD003214	–7.0	1719	SD002954	–7.0
1624	SD003426	–7.1	1672	SD001275	–7.0	1720	SD003427	–7.0
1625	SD003437	–7.1	1673	SD001405	–7.0	1721	SD002923	–7.0
1626	SD003099	–7.1	1674	SD001502	–7.0	1722	SD003000	–7.0
1627	SD000298	–7.1	1675	SD001504	–7.0	1723	SD002988	–7.0
1628	SD002619	–7.1	1676	SD001520	–7.0	1724	SD003110	–7.0
1629	SD003651	–7.1	1677	SD001470	–7.0	1725	SD003159	–7.0
1630	SD000353	–7.1	1678	SD000159	–7.0	1726	SD003152	–7.0
1631	SD003743	–7.1	1679	SD001662	–7.0	1727	SD000296	–7.0

Table S1. *Continued.*

No.	SuperDRUG2 Code	Standard Docking Score (kcal/mol)	No.	SuperDRUG2 Code	Standard Docking Score (kcal/mol)	No.	SuperDRUG2 Code	Standard Docking Score (kcal/mol)
1728	SD003157	–7.0	1740	SD003839	–7.0	1751	SD000450	–7.0
1729	SD000309	–7.0	1741	SD003860	–7.0	1752	SD000541	–7.0
1730	SD003484	–7.0	1742	SD003919	–7.0	1753	SD000456	–7.0
1731	SD003507	–7.0	1743	SD003898	–7.0	1754	SD000556	–7.0
1732	SD003499	–7.0	1744	SD003856	–7.0	1755	SD000613	–7.0
1733	SD003528	–7.0	1745	SD003770	–7.0	1756	SD000680	–7.0
1734	SD000340	–7.0	1746	SD006033	–7.0	1757	SD000766	–7.0
1735	SD003517	–7.0	1747	SD000053	–7.0	1758	SD000729	–7.0
1736	SD003520	–7.0	1748	SD006012	–7.0	1759	SD000736	–7.0
1737	SD003782	–7.0	1749	SD000047	–7.0	1760	SD000814	–7.0
1738	SD003787	–7.0	1750	SD000424	–7.0	1761	SD000739	–7.0
1739	SD003790	–7.0						
